# Fibroblast Nox2 (NADPH Oxidase-2) Regulates ANG II (Angiotensin II)–Induced Vascular Remodeling and Hypertension via Paracrine Signaling to Vascular Smooth Muscle Cells

**DOI:** 10.1161/ATVBAHA.120.315322

**Published:** 2020-10-15

**Authors:** Craig B. Harrison, Silvia Cellone Trevelin, Daniel A. Richards, Celio X.C. Santos, Greta Sawyer, Andrea Markovinovic, Xiaohong Zhang, Min Zhang, Alison C. Brewer, Xiaoke Yin, Manuel Mayr, Ajay M. Shah

**Affiliations:** 1 King’s College London British Heart Foundation Centre of Excellence, School of Cardiovascular Medicine and Sciences, United Kingdom (C.B.H., S.C.T., D.A.R., C.X.C.S., G.S., X.Z., M.Z., A.C.B., X.Y., M.M., A.M.S.).; 2 Department of Basic and Clinical Neuroscience, King’s College London, Maurice Wohl Clinical Neuroscience Institute, United Kingdom (A.M.).

**Keywords:** angiotensin II, fibroblast, hypertension, NADPH oxidase-2, vascular remodeling

## Abstract

The superoxide-generating Nox2 (NADPH oxidase-2) is expressed in multiple cell types. Previous studies demonstrated distinct roles for cardiomyocyte, endothelial cell, and leukocyte cell Nox2 in ANG II (angiotensin II)–induced cardiovascular remodeling. However, the in vivo role of fibroblast Nox2 remains unclear.

**Approach and Results:**

We developed a novel mouse model with inducible fibroblast-specific deficiency of Nox2 (fibroblast-specific Nox2 knockout or Fibro-Nox2KO mice) and investigated the responses to chronic ANG II stimulation. Fibro-Nox2KO mice showed no differences in basal blood pressure or vessel wall morphology, but the hypertensive response to ANG II infusion (1.1 mg/[kg·day] for 14 days) was substantially reduced as compared to control Nox2-Flox littermates. This was accompanied by a significant attenuation of aortic and resistance vessel remodeling. The conditioned medium of ANG II–stimulated primary fibroblasts induced a significant increase in vascular smooth muscle cell growth, which was inhibited by the short hairpin RNA (shRNA)-mediated knockdown of fibroblast Nox2. Mass spectrometric analysis of the secretome of ANG II–treated primary fibroblasts identified GDF6 (growth differentiation factor 6) as a potential growth factor that may be involved in these effects. Recombinant GDF6 induced a concentration-dependent increase in vascular smooth muscle cell growth while chronic ANG II infusion in vivo significantly increased aortic GDF6 protein levels in control mice but not Fibro-Nox2KO animals. Finally, silencing GDF6 in fibroblasts prevented the induction of vascular smooth muscle cell growth by fibroblast-conditioned media in vitro.

**Conclusions:**

These results indicate that fibroblast Nox2 plays a crucial role in the development of ANG II–induced vascular remodeling and hypertension in vivo. Mechanistically, fibroblast Nox2 may regulate paracrine signaling to medial vascular smooth muscle cells via factors, such as GDF6.

## Abstract 2

Supplemental Digital Content is available in the text.

**Highlights**

- Nox2 (NADPH oxidase-2) is expressed in multiple cell types and is involved in cardiovascular remodeling but the role of fibroblast Nox2 is incompletely defined.
- Mice with a fibroblast-specific deficiency of Nox2 display substantially reduced vascular remodeling and hypertension in response to chronic angiotensin II infusion.
- Fibroblast Nox2 regulates the paracrine release of GDF6 (growth differentiation factor 6) to exert growth effects on vascular smooth muscle cells.
- Fibroblast Nox2 plays a crucial role in the development of angiotensin II–induced vascular remodeling and hypertension.

**See accompanying editorial on page 711**

The arterial wall is a highly plastic structure with the capacity to adapt to changes in neurohumoral stimulation, blood pressure (BP), and flow during vascular diseases. This functional remodeling may involve all 3 layers of the vessel—the intima, media, and adventitia. Chronic activation of the renin-angiotensin system plays a key role in vascular remodeling and hypertension. ANG II (angiotensin II)–induced vascular remodeling is a major contributor to the increase in peripheral vascular resistance that characterizes hypertension.^1^ This remodeling involves a significant thickening of the vascular media which is driven by vascular smooth muscle cell (VSMC) hypertrophy, accompanied at a cellular level by increased nuclear ploidy and DNA synthesis.^2^ ANG II–induced vascular remodeling is also linked to an increased risk of other pathologies such as stroke.^3^ As such, inhibition of the renin-angiotensin system with angiotensin-converting enzyme inhibitors or ANG II receptor antagonists is a cornerstone of antihypertensive therapy.^4^

A large body of evidence implicates reactive oxygen species (ROS) production in the pathophysiology of ANG II–dependent remodeling and hypertension.^5,6^ ROS have complex cell and context-specific roles in this setting, including pathophysiologic redox signaling that contributes to VSMC growth, effects on vessel tone, and actions in other organs, such as the kidney and brain.^7–10^ Nox (NADPH oxidase) family proteins are major sources of vascular ROS in response to ANG II stimulation.^11^ Nox proteins mediate ROS production through catalytic subunits that transfer electrons from NADPH to molecular oxygen. Five distinct Nox enzymes (Nox1–5) with tissue-specific distribution and differing modes of activation have been identified, among which Nox1 and Nox2 are implicated in the development of ANG II–induced hypertension in rodents.^11^ Mice globally deficient in Nox1 display a blunted hypertensive response to chronic ANG II infusion,^8^ whereas transgenic mice with VSMC-targeted overexpression of Nox1 develop exaggerated vascular remodeling and hypertension.^12^ Mice with a global deficiency of Nox2 were also reported to develop less vascular remodeling and hypertension in response to chronic ANG II infusion.^13,14^ Although Nox1 is expressed primarily in VSMC, Nox2 is found in several cell types including endothelial cells, fibroblasts, inflammatory cells, and microglia.^11^ Recent studies have shown that Nox2 in endothelial cells, microglial cells, and leukocytes has distinct effects on ANG II–induced pathology and hypertension.^10,15–17^ For example, endothelial Nox2 activation is important in augmenting the early hypertensive response to chronic ANG II infusion, whereas myeloid cell Nox2 was found to modulate basal BP by reducing nitric oxide bioavailability.^16^ The potential role of Nox2 in fibroblasts is, however, unclear.

In this study, we developed a novel mouse model with fibroblast-specific deficiency of Nox2 to investigate the role of fibroblast Nox2 in ANG II–induced hypertension. The results show that fibroblast Nox2 plays a major role in the development of chronic ANG II–induced vascular remodeling and hypertension. These effects are governed at least in part by paracrine signaling involving the production and release of GDF6 (growth differentiation factor 6) by Nox2-activated fibroblasts to induce VSMC growth.

## Methods

The authors declare that all supporting data are available within the article (and its Data Supplement).

### Animals

Animal studies were performed in accordance with UK Home Office Guidance on the Operation of the Animals (Scientific Procedures) Act, 1986, and with institutional ethical approval. *Nox2*^fl/fl^ mice on a C57BL/6J background were described recently.^16^ Fibroblast-specific Nox2 knockout (Fibro-Nox2KO) mice were generated by first crossing female *Nox2*^fl/fl^ mice with male transgenic animals expressing a tamoxifen-inducible Cre recombinase under the control of a fibroblast-specific regulatory enhancer element of the mouse *Col1α2* gene (*Col1α2*CreER-Tg)^18^ also on a C57BL/6J background. Male *Col1α2*CreER/*Nox2*^fl/y^ mice were compared with littermate male mice expressing *Nox2*^fl/y^ without *Col1α2*CreER (“control” *Nox2*-Flox mice). We used male mice because this simplified the breeding strategy (as *Nox2* is located on the X chromosome) and also to minimize estrogen-dependent fluctuations in response to ANG II.^19^ Both groups were treated with tamoxifen (50 mg/kg IP, 10 days) at the age of 7 to 8 weeks to induce fibroblast-specific Nox2 deficiency in *Col1α2*CreER/*Nox2*^fl/y^ mice (ie, Fibro-Nox2KO mice). Treatment with tamoxifen had no effect on cardiac function or BP in *Col1α2*CreER-Tg mice (data not shown). Some *Col1α2*CreER-Tg mice were also crossed ROSA26R-tdTomato^fl/fl^ mice (Jackson Labs).

### In Vivo Procedures

Ambulatory BP was measured by radiotelemetry.^16^ Telemeters (model TA11PA-C10, Data Sciences International, Netherlands) were implanted subcutaneously under isoflurane anesthesia, with the transducer inserted via the left carotid artery into the aorta. Flunixin-meglumine (5 mg/kg SC) was administered for analgesia. Measurements were made after a minimum 1-week recovery period. Analyses were performed using Dataquest ART software. ANG II (1.1 mg/[kg·day]) or vehicle were infused via subcutaneous osmotic minipumps (model 1002; Alzet, Cupertino, CA), implanted under 2% isoflurane. Echocardiography was performed under 1% isoflurane anesthesia using a Vevo 2100 System (VisualSonics, Toronto, Canada) equipped with a 22 to 55 MHz linear-array transducer.^16^

### Perfusion Fixation and Histology

Mice were terminally anesthetized with a pentobarbitone overdose and perfused via the left ventricle at ≈110 mm Hg, first with saline and then 4% paraformaldehyde. Aorta, carotid arteries, and hearts were dissected and frozen in optimal cutting temperature compound before cryosectioning into 10 μm sections. Sections were stained with hematoxylin and eosin, dehydrated in ethanol, and cleared in xylene before cover-slipping with distyrene plasticizer xylene mounting solution. Images were captured on a Zeiss AXIOSKOP microscope with ×5 and ×10 objectives and analyzed using Volocity software (PerkinElmer, United Kingdom).

### Immunofluorescence

Aorta was dissected, taking care not to lose the adventitia and frozen in optimal cutting temperature compound before making 12 μm cryosections. Tissues were fixed with 4% paraformaldehyde, permeabilized with 0.2% Triton, and blocked with 3% BSA (bovine serum albumin) and 1.5% goat serum for 30 minutes each. Sections were incubated overnight with anti-Nox2 (2.5 µg/mL, Catalog no. 611415; BD Biosciences), anti–smooth muscle actin (4 µg/mL, Catalog no. A2447; Sigma), anti-vimentin (5 µg/mL, Catalog no. Ab8069; Abcam), WGA (wheat germ agglutinin)-rhodamine (20 µg/mL, Catalog no. RL1022S; Vector laboratories), anti-VE-cadherin (vascular endothelial cadherin; 10 µg/mL, Catalog no. 138002; Biolegend), anti-Ki67 (1:200, Catalog no. NB-600-1252; Novus Biologicals), or anti-GDF6 (10 µg/mL, Abcam, Catalog no. Ab73288) antibodies before washing and incubating with an Alexa-594 conjugated fluorescent secondary antibody (anti-rabbit for GDF6; anti-mouse for smooth muscle actin or Nox2). Nuclei were stained with 4′-6-diamidino-2-phenylindole (Sigma). Images were captured on an Olympus 1X81-2 fluorescent microscope using a ×40 objective or a Leica confocal TCS SP5 microscope using a ×63 objective. Analyses of mean fluorescence intensity and co-localization were performed using Image J software (v1.0 Mac OS X; National Institutes of Health).

Brains were fixed with 4% paraformaldehyde for 16 hours and embedded in optimal cutting temperature compound, then cryosectioned into 25 μm coronal slices. The tissue was permeabilized with 0.25% Triton and blocked with 1% BSA and 10% horse serum. After overnight incubation with anti-Nox2 antibody and washing, slices were incubated with Alexa-555 anti-rabbit antibody. Imaging was performed using an Olympus VS120 slide scanner and Nox2 optical density in the subfornical organ was analyzed using Image J.

### Immunoblotting

Sections of aorta were snap-frozen in liquid nitrogen, crushed with a micropestle, and protein was extracted using Laemmli buffer and sonication. Bone marrow cells were flushed in PBS (phosphate-buffered saline), centrifuged at 500*g* for 5 minutes, and the pellet solubilized in RIPA (radioimmunoprecipitation assay) buffer (50 mmol/L Tris-HCl pH 7.2, 150 mmol/L NaCl, 2 mmol/L EDTA, 1%Triton). The conditioned medium from ANG II–stimulated fibroblasts was dried and the pellet dissolved in Laemmli buffer. Immunoblots were performed using 4% to 12% sodium dodecyl sulfate (SDS)-polyacrylamide gels and standard methods. Antibodies used were: Nox2 (0.25 µg/mL, Catalog No. 611415; BD Biosciences, United Kingdom), β-actin (0.67 µg/mL; Sigma, United Kingdom), GDF6 (1µg/mL; Abcam), and HTRA1 (serine protease HTRA1; 2 µg/mL, Abcam ab38611, United Kingdom). An LI-COR Odyssey system (LI-COR, NE) and secondary antibodies were used to reveal and quantify immunoreactive bands. Total protein was determined by an LI-COR Revert 700 staining Kit for Western Blot Normalization (Catalog no. P/N 926-11016) or Ponceau staining.

### Real-Time Polymerase Chain Reaction

Whole aortas were homogenized in TRIzol reagent (ThermoFisher) for RNA extraction. Reverse transcription was performed using standard methods and polymerase chain reaction was performed on an Applied Biosystems 7000 system (Applied Biosystems, United Kingdom) using SYBR Green and the comparative *C*_t_ (threshold cycle) method, with cytoskeletal β-actin levels used for normalization. Forward and reverse primers were as follows (all 5′-3):

β-actin Forward: CTGTCGAGTCGCGTCCACCC; Reverse: ATGCCGGAGCCGTTGTCGAC; TGF (transforming growth factor)-β1 Forward: TGGAGCAACATGTGGAACTC; Reverse: GTCAGCAGCCGGTTACCA; TGF-β2 Forward: AGGAGGTTTATAAAATCGACATGC; Reverse: TAGAAAGTGGGCGGGATG; TGF-β3 Forward: GCAGACACAACCCATAGCAC; Reverse: GGGTTCTGCCCACATAGTACA; GDF6 Forward: TAGCTTCCTCTGGGATTTGC, Reverse: GAGGAGGAGGACGAGGAGAT; LOXL2 Forward: GGAGAACAAGGCATCACCAT, Reverse: GTTGGGGTTAATGCAAACCTG; SFRP1 Forward: CAGTTGTGGCTTTTGCATTG, Reverse: GAGGGAAGGGAGAGGGTT; HTRA1 (HtrA serine peptidase 1) Forward: CATTGAAGTCATTCCTGACAC, Reverse: TGTCCGTTGATGCTGATG; NOX2 Forward: CCAACTGGGATAACGAGTTCAA, Reverse: TCAGGGCCACACAGGAAAA; p22^phox^ Forward: TGGACGTTTCACACAGTGGTA, Reverse: TGGACCCCTTTTTCCTCTTTC; p67^phox^ Forward: AAGCTGTTTGCCTGTGAGGT, Reverse: CTTCATGTTGGTTGCCAATG; p47^phox^ Forward: AGAGTCGCCAGGGCACTCT, Reverse: TCTTCGCCTGGCTGTCAGT; Nox4 Forward: CCGGACAGTCCTGGCTTATC, Reverse: TGCTTTTATCCAACAATCTTCTTTT; Nox1 Forward: CGTGAAAAGATGACCCAGATCA, Reverse: TGGTACGACCAGAGGCATACAG.

### Cells

Primary cardiac fibroblasts were isolated, as previously described.^20^ They were seeded in 0.05% gelatin-coated culture flasks containing DMEM (Dulbecco’s modified Eagle’s medium; Sigma, D6546) supplemented with 10% FCS (fetal calf serum), penicillin, and streptomycin and maintained at 37 °C in humidified 5% CO_2_. Fibroblasts were used at passage 1. Mouse aortic VSMC were isolated and maintained in 10% FCS and 1% NAAA (α-napthaleneacetic acid) containing medium (Sigma: D5671) and studied at passage 2 to 3.^21^ VSMC were seeded at 10 000 cells per well in 24 well plates or 40 000 cells per well in 6 well plates 24 hours before the start of an experiment. Aortic fibroblasts were purchased from Cell Biologics (C57BL/6 mouse primary aortic fibroblasts; Catalog no. C57-6075) and cultured in complete fibroblast medium (Cell Biologics; Catalog no. M2267).

### VSMC Growth Assays

Fibroblasts were treated with 200 nmol/L ANG II or vehicle in 0.1% FCS medium for 48 hours, after which the medium was removed and centrifuged at 310*g* for 5 minutes. In some experiments, fibroblasts were first transduced with adenoviruses expressing shRNA (short hairpin RNA) targeted against Nox2 (Ad.shNox2) or adenoviruses expressing shRNA targeted against a GFP (green fluorescent protein) control,^10^ then treated with ANG II and the conditioned medium prepared. Some fibroblasts were transfected with siRNA (silencing RNA) against GDF6 or scrambled siRNA (Catalog no. MBS8236943; MyBiosource) using lipofectamine 2000 24 hours before stimulation with ANG II. VSMC seeded onto glass cover-slips were incubated with fibroblast-conditioned medium for 48 hours, then the cells were fixed and prepared for staining. In some experiments, VSMC were treated with recombinant GDF6 (PeproTech Human BMP-13/CDMP-2) for 24 hours before preparing for staining. Cells were incubated with anti-ki67 (Vector: 1:100) and anti–smooth muscle actin (Sigma: 1:1000) antibodies for 1 hour at room temperature or were first treated with 10 µmol/L BrdU (bromodeoxyuridine; Sigma) per well and then incubated with anti-BrdU (10 µg/mL, Catalog no. ab152095; Abcam) and anti–smooth muscle actin (4 µg/mL, Catalog no. A2447; Sigma) antibodies. Cells were then washed and incubated with Alexa 568 conjugated anti-rabbit and Alexa 488 conjugated anti-mouse secondary antibodies, then 4′-6-diamidino-2-phenylindole before mounting onto microscope slides in FluoromountTM aqueous mounting medium (Catalog no. F4680; Sigma). Images were captured on an Olympus 1X81-2 fluorescent microscope and analyzed using Volocity software or a Leica TCS SP5 confocal microscope and analyzed using Image J.

### Secretome Analysis

Primary fibroblasts in T25 culture flasks were infected with Ad.shNox2 or adenoviruses expressing shRNA targeted against a GFP control, then treated with ANG II (200 nmol/L) or vehicle in FCS-free medium for 48 hours. The medium was then removed, spun at 15 000*g* for 10 minutes, and the supernatant was collected and frozen at −80 °C. Proteomic analysis of the supernatants was performed by mass spectrometry as previously described.^22^ For immunoblot analyses, the conditioned medium was lyophilized before resuspending in Laemmli buffer.

### TGF-β ELISA

Primary cardiac fibroblasts were treated with ANG II (200 nmol/L) or vehicle for 2 days, then the ELISA (TGF-β1 ELISA kit, Catalog no. 88-8350-22; Affymetrix) was performed on the conditioned medium.

### ROS Assay

Dihydroethidium fluorescence was used to estimate ROS production in aorta. For this, 7-µm thick tissue sections were incubated with 3 µmol/L dihydroethidium in Hank’s without phenol red, with diethylenetriamine-pentaacetic acid (100 µmol/L) for 30 minutes. Images were then obtained immediately on an Olympus 1X81 inverted epifluorescence microscope.

The dihydroethidium oxidation product, 2-hydroxyethidium, was quantified in aorta and cultures of fibroblasts as previously described.^16^ Aortae from mice chronically infused with ANG II or fibroblasts stimulated with ANG II (200 nmol/L, 4 hours) were incubated with dihydroethidium (100 μM) for 30 minutes at 37 °C and then lyophilized in acetonitrile. Quantification of 2-hydroxyethidium was performed by high-performance liquid chromatography (HPLC).

### Renal Function

Mice were challenged with 1 mL IP PBS, and the urine collected hourly in a metabolic cage for 4 hours.^16^ Urine osmolarity, sodium, and potassium concentrations were analyzed on an Advia 2400 Chemistry System (Siemens AG, Germany).

### Statistics

Data are expressed as mean±SEM. The Kolmogorov-Smirnov normality test (with Dallal-Wilkinson-Lilliefor corrected *P* value) was used to test normality of the samples. Comparisons were made by Student unpaired *t* tests, 1-way ANOVA, or 2-way ANOVA followed by Tukey post hoc test or a nonparametric Kruskal-Wallis followed by Dunn post-test, as appropriate. Data were analyzed on GraphPad Prism v6. *P*<0.05 was considered significant.

## Results

### Fibroblast-Specific Deletion of Nox2 In Vivo Has No Effect on Basal BP

**Figure 1.**
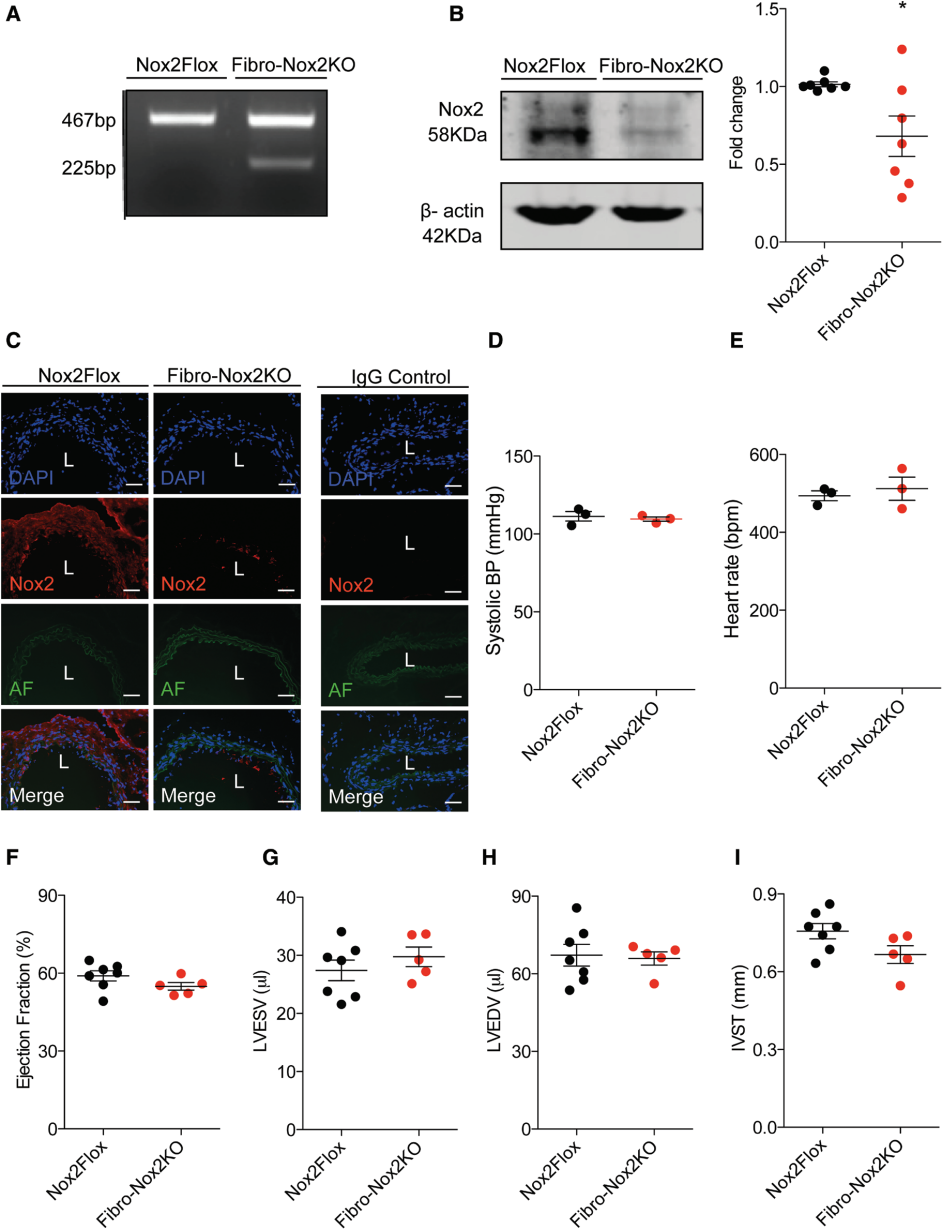
**Basal phenotype of Fibro-Nox2KO mice.** **A**, Polymerase chain reaction showing Cre-mediated recombination in Fibro-Nox2KO (fibroblast-specific Nox2 knockout) aorta. **B**, Nox2 protein levels in aorta from Fibro-Nox2KO and control (Nox2Flox) mice. A representative immunoblot is shown along with mean data. n=7 per group. *P<0.05, unpaired Student *t* test. **C**, Immunofluorescence for Nox2 (Red) in aortic sections from Fibro-Nox2KO and control mice. Blue, nuclei stained with 4′-6-diamidino-2-phenylindole (DAPI); green, autofluorescence (AF) of the vessel wall. Scale bars, 50 µm. **D**, Systolic blood pressure (BP) and (**E**) heart rate measured by ambulatory telemetry in Fibro-Nox2KO and control mice (n=3 per group). **F–I**, Cardiac structure and function assessed by echocardiography. n=5–7 per group. IVST indicates interventricular septal thickness; L, vessel lumen; LVEDV, left ventricular end-diastolic volume; LVESV, left ventricular end-systolic volume; and Nox2, NADPH oxidase-2.

Inducible deletion of fibroblast Nox2 was achieved by intraperitoneal injection of tamoxifen in adult *Col1α2*CreER/*Nox2*^fl/y^ mice, to generate Fibro-Nox2KO animals. Fibro-Nox2KO mice showed evidence of Cre-mediated recombination in DNA isolated from aorta (Figure 1A), as well as a significant decrease in Nox2 protein levels by Western blot (Figure 1B). There were no significant differences in the mRNA levels of p22^phox^, p47^phox^, p67^phox^, Nox1, or Nox4 between Fibro-Nox2KO and control *Nox2*-Flox mouse aortae but mRNA levels of Nox2 were significantly reduced (Figure IA through IF in the Data Supplement). Immunofluorescence of aortic sections revealed a marked reduction in Nox2 immunostaining in the adventitia of the Fibro-Nox2KO group compared to control mice, whereas endothelial Nox2 staining was preserved (Figure 1C). Fibro-Nox2KO mice did not show differences in Nox2 protein levels in bone marrow cells as compared to *Nox2*-Flox mice (Figure IIA in the Data Supplement). To further assess the cell-specificity of the *Col1α2*CreER approach, we crossed *Col1α2*CreER mice with ROSA26R-tdTomato^fl/fl^ mice, in which expression of tdTomato fluorescence is dependent on Cre-mediated excision of a STOP codon. In the progeny, tdTomato fluorescence would, therefore, be expressed in cells targeted by the *Col1α2* promoter and expressing Cre recombinase. Using this approach, tdTomato fluorescence in heart sections overlapped with vimentin staining in the adventitia of coronary vessels (used as a fibroblast marker) but not at the lumen in the endothelium (Figure IIB in the Data Supplement). Furthermore, there was no overlap between tdTomato fluorescence and VE-cadherin, used as an endothelial marker (Figure IIC in the Data Supplement). Taken together, these results indicate the *Col1α2*CreER approach results in fibroblast-specific genetic targeting.

There was no difference between Fibro-Nox2KO and control mice in body or organ weights and no obvious basal phenotype (data not shown). Ambulatory systolic BP, diastolic BP, and heart rates were similar in the two groups under baseline conditions (Figure 1D). Cardiac dimensions and function assessed by echocardiography were also unaltered in Fibro-Nox2KO mice compared with control (Figure 1F through 1I).

### ANG II–Induced Hypertension and Vascular Remodeling Are Inhibited in Fibro-Nox2KO Mice

**Figure 2.**
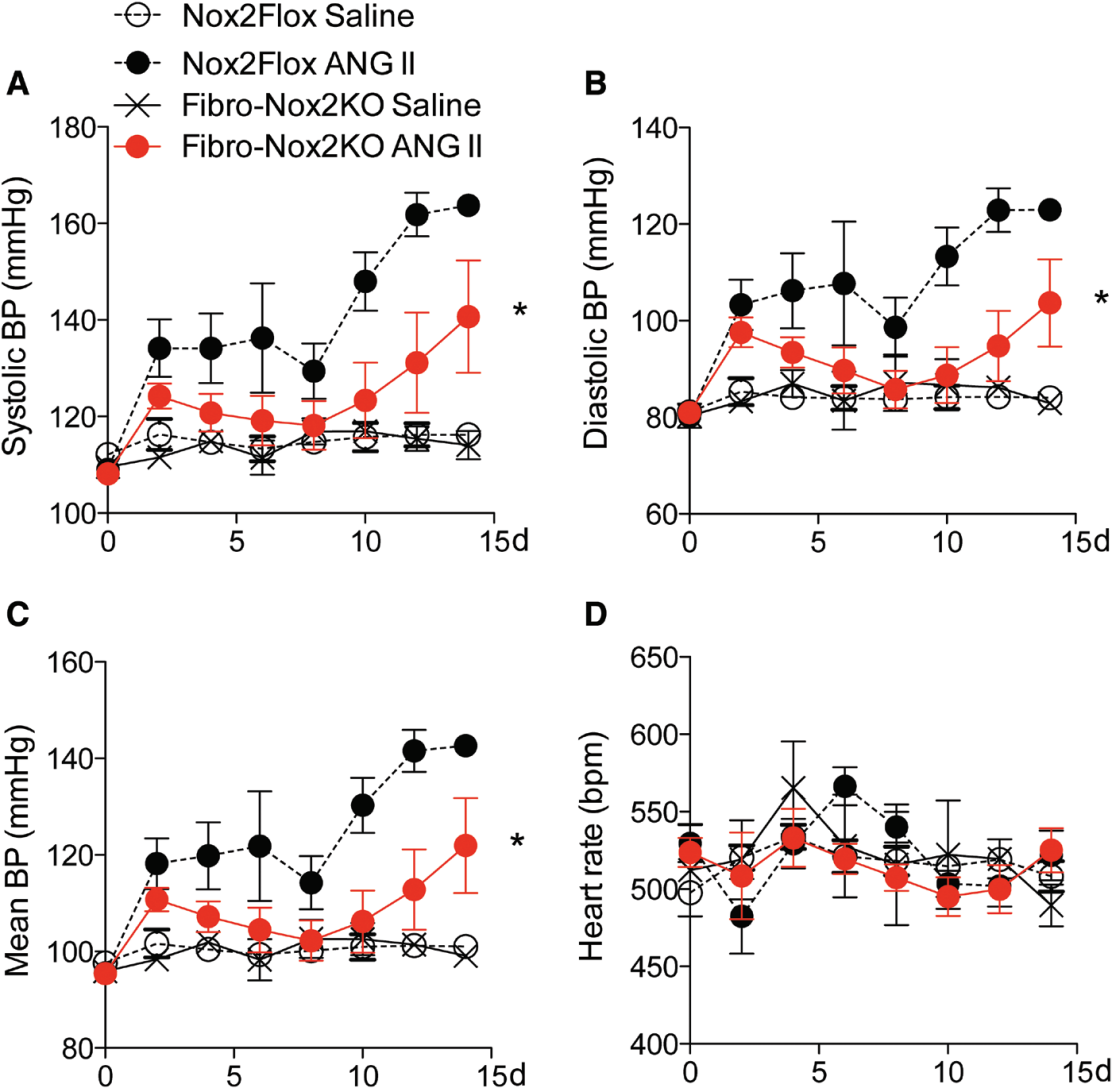
**ANG II (angiotensin II)–induced hypertension is inhibited in Fibro-Nox2KO mice.** Telemetric ambulatory blood pressure (BP) in fibroblast-specific Nox2 knockout (Fibro-Nox2KO) and matched control (*Nox2*Flox) mice treated with chronic ANG II infusion or saline. **A**, Systolic BP (BP); (**B**) diastolic BP; (**C**) mean arterial BP; (**D**) heart rate. 2-way repeated-measures ANOVA, n=3–7 per group. Nox2 indicates NADPH oxidase-2. *P<0.05 for ANG II–treated FibroNox2KO vs ANG II–treated Nox2Flox.

We next tested the BP response to chronic 2-week ANG II infusion in Fibro-Nox2KO and control mice, using ambulatory telemetry. Although control mice developed a significant increase in systolic, mean, and diastolic BP by the end of 2-week infusion, this response was markedly blunted in Fibro-Nox2KO animals (Figure 2A through 2C). There was no difference in heart rate between groups (Figure 2D). Aortic sections from Fibro-Nox2KO mice infused with ANG II showed a lower dihydroethidium fluorescence signal than those from control mice under the same treatment, and levels of the dihydroethidium oxidation product 2-hydroxyethidium measured by HPLC in aortic homogenate were also significantly reduced (Figure IIIA and IIIB in the Data Supplement). Moreover, fibroblasts isolated from Fibro-Nox2KO mice showed lower 2-hydroxyethidium levels than cells from Nox2-Flox mice after in vitro stimulation with ANG II (Figure IIIC in the Data Supplement).

**Figure 3.**
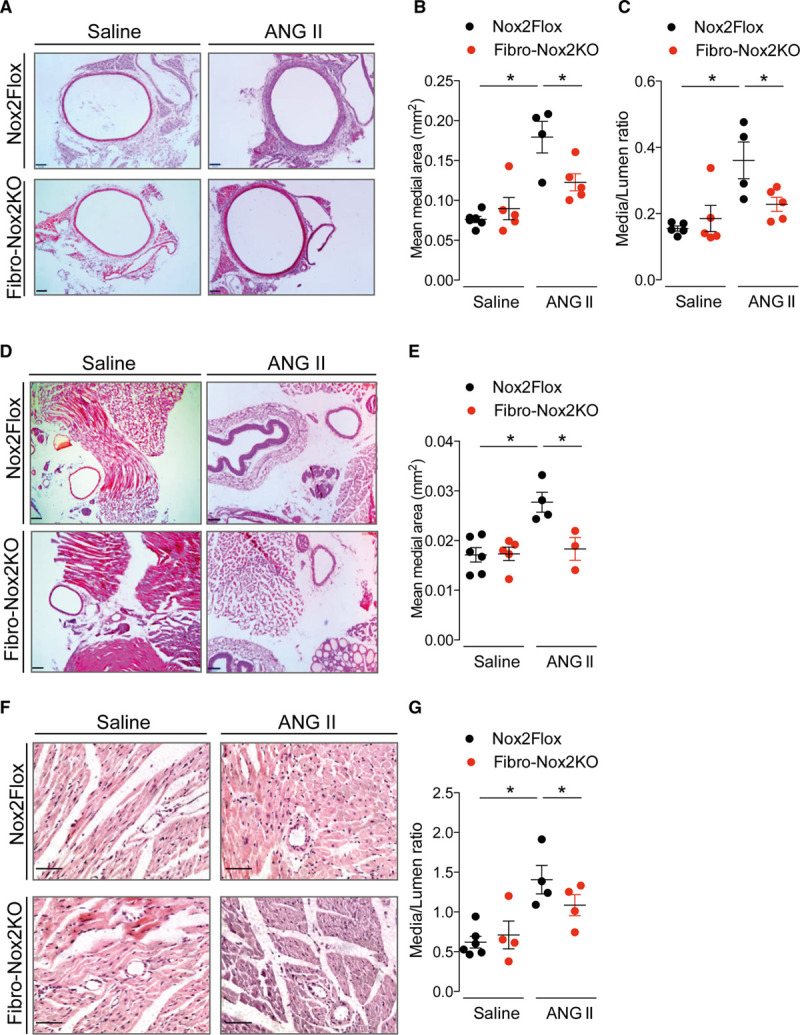
**ANG II (angiotensin II)–induced vascular remodeling is inhibited in Fibro-Nox2KO mice.** Control (*Nox2*Flox) and fibroblast-specific Nox2 knockout (Fibro-Nox2KO) mice were chronically treated with ANG II or vehicle, then perfusion-fixed. **A**, Representative aortic sections. Scale bars, 100 µm. **B**, Mean aortic medial area and (**C**) media/lumen ratio; n=4–6 aortae per group. **D**, Representative sections of carotid artery. Scale bars, 100 µm. **E**, Mean medial area of carotid artery sections; n=3–6 arteries per group. **F**, Representative myocardial sections showing small septal arteries. Scale bars, 50 µm. **G**, Mean media/lumen ratio of septal arteries; n=4–6 hearts per group and 5 sections per heart. 1-way ANOVA followed by Tukey post-test. Nox2 indicates NADPH oxidase-2. *P<0.05.

Assessment of renal function with an intraperitoneal saline challenge test revealed no significant differences in urine osmolarity, sodium, or potassium concentration in Fibro-Nox2KO mice compared to controls (Figure IV in the Data Supplement). However, aortic sections from control mice that had received 2-week ANG II infusion showed a significant increase in medial area and media/lumen ratio, whereas these changes were inhibited in Fibro-Nox2KO mice (Figure 3A through 3C). There were no differences in medial area between vehicle-treated groups. To assess whether similar changes occurred in smaller resistance arteries, we analyzed carotid arteries and small septal arteries in myocardial sections. A similar inhibition of medial thickening to that observed in the aorta was also found in carotid arteries (Figure 3D through 3E) and septal arteries (Figure 3F through 3G) of ANG II–treated Fibro-Nox2KO mice compared with controls.

Since Nox2 activation in the subfornical organ of the brain is also implicated in mediating the hypertension induced by ANG II infusion,^10^ we evaluated Nox2 expression in the subfornical organ by immunostaining of brain sections. These studies showed that Nox2 immunostaining was similar in FibroNox2KO and Nox2-Flox brain subfornical organ (Figure V in the Data Supplement). Brain sections from global Nox2 KO mice and sections stained with nonspecific IgG were used as controls. Therefore, Nox2 levels in the subfornical organ do not appear to be altered in FibroNox2KO mice.

### Nox2 Deficiency in Fibroblasts Inhibits ANG II–Induced Vascular Smooth Muscle Growth

**Figure 4.**
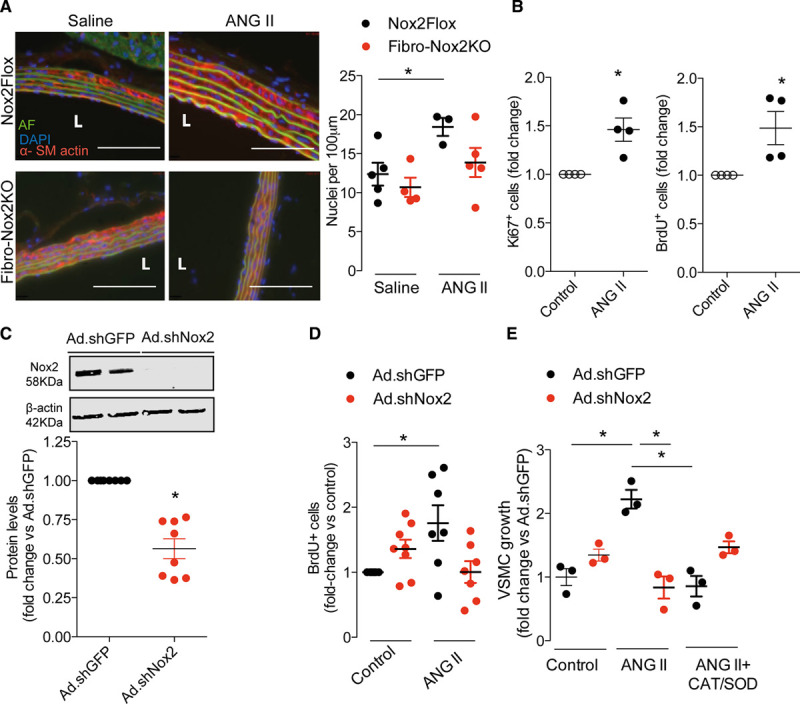
**Fibroblast Nox2 (NADPH oxidase-2) is required for ANG II (angiotensin II)–induced vascular smooth muscle cell (VSMC) growth.** **A**, Increased VSMC ploidy in aortic media of ANG II–treated control (*Nox2*Flox) vs fibroblast-specififc Nox2 knockout (Fibro-Nox2KO) mice. Representative aortic sections examined by immunofluorescence are shown to the **left**. Red, α-smooth muscle actin; blue, 4′-6-diamidino-2-phenylindole (DAPI; nucleus); green, autofluorescence (AF). Scale bars, 50 µm. Mean data for number of DAPI-positive nuclei/100 μm of media shown to the **right**; n=3–5 aortae per group. **B**, Effect of ANG II–conditioned cardiac fibroblast medium on VSMC growth, assessed by Ki67 staining or BrdU (bromodeoxyuridine) incorporation, respectively; n=4 per group. **C**, Knockdown of Nox2 in cardiac fibroblasts by adenoviruses expressing shRNA (short hairpin RNA) targeted against Nox2 (Ad.shNox2) compared with adenoviruses expressing shRNA targeted against GFP (green fluorescent protein) control (Ad.shGFP). Representative immunoblot at the **top** shows Nox2 knockdown; mean data for Nox2 protein levels are shown below; n=8 per group. **D**, Conditioned medium from fibroblasts treated with Ad.shNox2 has reduced growth activity on VSMC as assessed by BrdU incorporation; n=7–9 per group. **E**, Preincubation of fibroblasts with SOD (PEG-superoxide dismutase) and CAT (PEG-catalase) prevents the effect of conditioned media on VSMC growth. n=3 per group. Unpaired Student *t* test (**B** and **C**) or 1-way ANOVA followed by Tukey post-test (**A** and **D**) or Kruskal-Wallis followed by Dunn post-test (**E**). *P<0.05. L indicates vessel lumen.

The increase in vascular medial thickness after ANG II infusion is thought to reflect an increase in VSMC growth and hypertrophy.^1,2^ To investigate whether deficiency of Nox2 in fibroblasts affects ANG II–induced VSMC growth, we first assessed medial VSMC growth in aortic sections by counting the number of nuclei of smooth muscle actin-positive cells in the medial layer (since VSMC hypertrophy is accompanied by increased ploidy^2^). This analysis revealed a nearly 50% increase in the number of nuclei in Ang II–treated compared to vehicle-treated control mice (Figure 4A). However, aortic sections of ANG II–treated Fibro-Nox2KO mice showed a significantly smaller increase in number of nuclei, suggesting that deficiency of Nox2 in fibroblasts affects VSMC growth during ANG II stimulation.

To more directly investigate a potential effect of fibroblasts on VSMC growth, we next assessed the response of cultured VSMC to incubation with the conditioned medium of primary cardiac fibroblasts treated with ANG II or vehicle. VSMC growth was assessed by the quantification of ki67 staining or BrdU incorporation (which assess DNA synthesis) and was found to be significantly increased by incubation with the conditioned medium of ANG II–treated fibroblasts (Figure 4B). To assess the role of fibroblast Nox2 in this response, fibroblasts were transduced with Ad.shNox2 or adenoviruses expressing shRNA targeted against a GFP control before treatment with ANG II and the collection of conditioned medium. Fibroblast Nox2 protein levels were substantially reduced by infection with Ad.shNox2 (Figure 4C). We found that the increase in VSMC growth induced by fibroblast-conditioned medium was substantially inhibited by the knockdown of Nox2 (Figure 4D). Similar results were obtained with conditioned media obtained from aortic fibroblasts (Figure VI in the Data Supplement). The incubation of fibroblasts with catalase and superoxide dismutase before collection of conditioned media also significantly reduced the ability of the media to induce VSMC growth in vitro (Figure 4E). These results suggest that Nox2 modulates the paracrine effects of fibroblasts on VSMC growth in a redox-dependent manner.

### Identification of GDF6 as a Nox2-Dependent Growth Factor Secreted by Fibroblasts

The treatment of fibroblast-conditioned medium with catalase did not alter its effects on VSMC growth (Figure VII in the Data Supplement), indicating that the response does not depend upon the presence of hydrogen peroxide in the medium (ie, does not involve the direct effects of hydrogen peroxide). To assess whether the fibroblast-conditioned medium contains factors that may mediate its effects on VSMC growth, we analyzed the secretome of ANG II–treated fibroblasts by mass spectrometry. A total of 460 proteins were identified of which 201 were predicted to be extracellular and potentially secreted, using DAVID bioinformatic software 6.7 (Figure VIIIA and VIIIB in the Data Supplement). To narrow down fibroblast-specific secreted factors, these proteins were compared to the mouse VSMC secretome profile^22^ using VENNY (http://bioinfogp.cnb.csic.es/tools/venny/). This analysis identified 121 fibroblast-secreted proteins that are not abundantly secreted by primary mouse VSMC (Figure VIIIC in the Data Supplement). We then performed gene ontology enrichment on these proteins and looked for those that have previously been reported to have growth properties (Figure IX in the Data Supplement). Based on this analysis, 6 proteins were highlighted for further investigation—namely, SFRP1 (secreted frizzled-related protein 1), LOXL2 (lysyl oxidase homolog 2), HTRA1, GDF6, TGF-β1, and TGF-β3.

**Figure 5.**
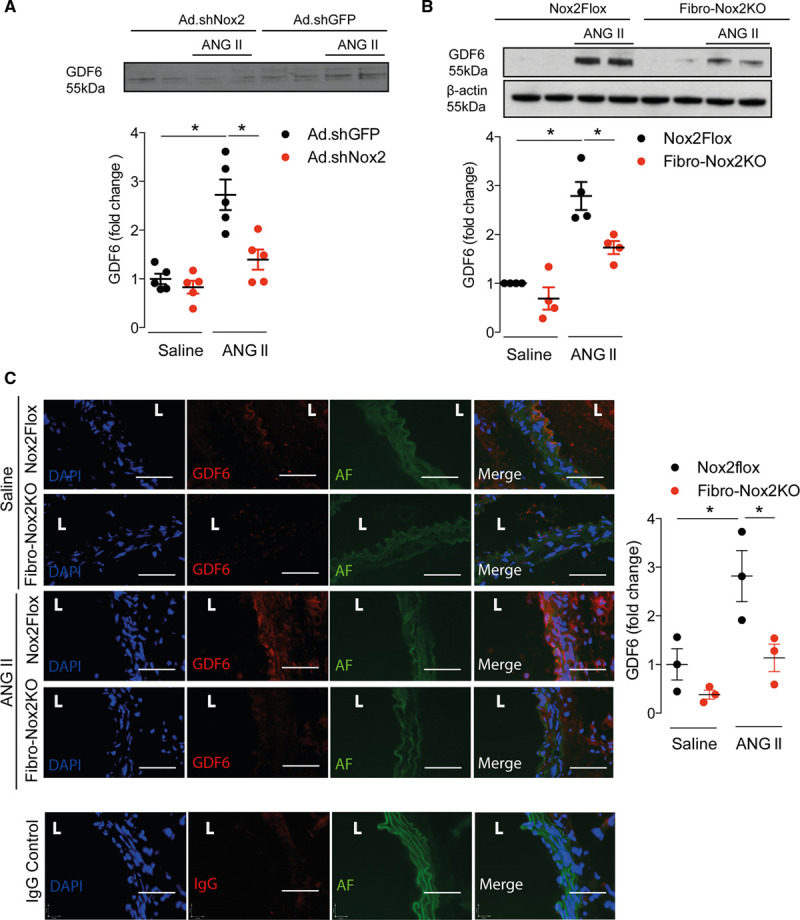
**Nox2 (NADPH oxidase-2)-dependent upregulation of GDF6 (growth differentiation factor 6).** **A**, GDF6 levels in conditioned medium of primary cardiac fibroblasts infected with adenoviruses expressing shRNA (short hairpin RNA) targeted against Nox2 (Ad.shNox2) or adenoviruses expressing shRNA targeted against a GFP (green fluorescent protein) control (Ad.shGFP; control) and treated with ANG II (angiotensin II) or vehicle. Representative immunoblot shown at the **top** and mean data at the **bottom**; n=3 per group. Ponceau staining of the membrane is shown in Figure XI in the Data Supplement. **B**, GDF6 protein levels in aorta from Fibro-Nox2KO or matched control (*Nox2*Flox) mice chronically treated with ANG II or saline vehicle. Representative immunoblot shown at the **top** and mean data at the **bottom**; n=4 per group. β-actin was used as a loading control. Densitometry values for GDF6-specific bands were divided by the corresponding densitometry of Ponceau bands in **A** and β-actin bands in **B**. Arbitrary units were calculated by normalizing data vs the control group (ie, fold-change). **C**, GDF6 immunostaining in aorta from Fibro-Nox2KO and control mice treated with ANG II or saline. Representative images are shown on the **left** and mean fluorescence intensity on the **right**. GDF6, red; 4′-6-diamidino-2-phenylindole (DAPI), blue; autofluorescence (AF), green. Scale bars=50 µm. 1-way ANOVA followed by Tukey post-test. L indicates vessel lumen. *P<0.05.

We quantified the ANG II–induced changes in gene expression of these proteins in fibroblasts transduced with Ad.shNox2 or adenoviruses expressing shRNA targeted against a GFP control. ANG II significantly increased the mRNA levels of GDF6, TGF-β1, and HTRA1 in control fibroblasts, but this response was markedly blunted in Nox2-deficient fibroblasts (Figure X in the Data Supplement). There were no significant differences between groups in SFRP1 or LOXL2. Based on this analysis, we quantified protein levels of GDF6, HTRA1, and TGF-β1 in the conditioned medium of ANG II or saline-treated fibroblasts deficient in Nox2 as compared to control fibroblasts. We did not find differences in HTRA1 levels between groups by immunoblotting (Figure XI in the Data Supplement) and could not detect TGF-β1 in fibroblast-conditioned media using ELISA (Figure XII in the Data Supplement). However, GDF6 protein levels in the control fibroblast secretome were significantly increased by ANG II treatment, and this response was inhibited in Nox2-deficient fibroblasts (Figure 5A and Figure XI in the Data Supplement). To confirm the in vivo relevance of this finding, GDF6 levels were assessed in aorta of Fibro-Nox2KO and control mice with and without chronic ANG II treatment. Western blot analysis showed that ANG II treatment resulted in a significant increase in aortic GDF6 levels in control mice but this was markedly blunted in Fibro-Nox2KO animals (Figure 5B). Immunostaining of aortic sections also confirmed a substantial increase in GDF6 levels in ANG II–treated control mice, with a much smaller increase in Fibro-Nox2KO aorta (Figure 5C).

**Figure 6.**
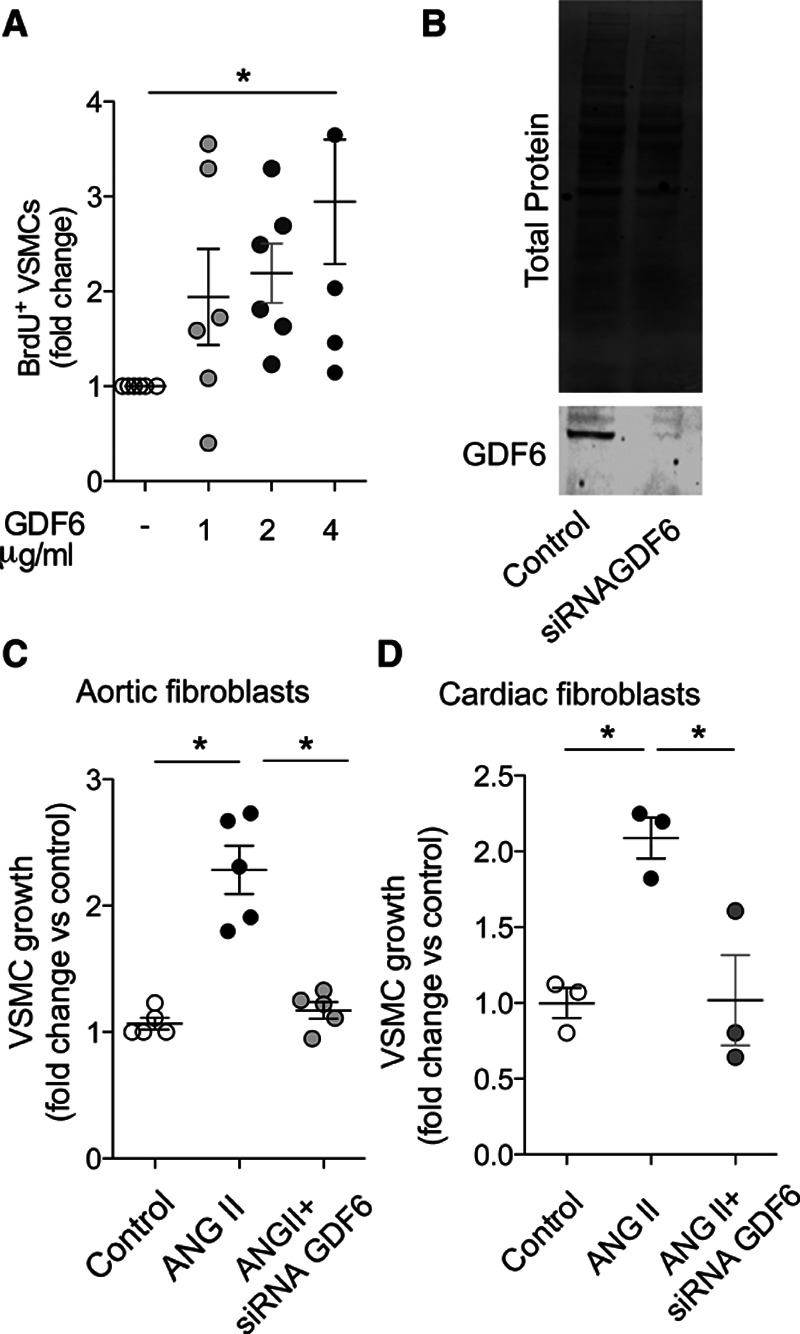
**GDF6 (growth differentiation factor 6) induces vascular smooth muscle cell (VSMC) growth.** **A**, Concentration-dependent effect of recombinant GDF6 (0–4 μg/mL) on BrdU (bromodeoxyuridine) incorporation in VSMC; n=4–6 per group. **B**, Representative Western blot for GDF6 expression after silencing using siRNA (silencing RNA) against GDF6 or scrambled control (control) in primary cardiac fibroblasts. **C** and **D**, VSMC growth assessed by Ki67 staining after incubation with conditioned media of aortic (**C**) or cardiac (**D**) fibroblasts treated with ANG II (angiotensin II). Some fibroblasts were treated with siGDF6 (silencing RNA against GDF6) to reduce GDF6 levels. 1-way ANOVA followed by Tukey post-test (**A** and **C**) or Kruskal-Wallis followed by Dunn post-test (**D**). *P<0.05.

Next, the effects of recombinant GDF6 were tested on cultured VSMC. GDF6 induced a dose-dependent increase in VSMC growth as assessed by BrdU incorporation (Figure 6A). Additionally, silencing GDF6 in aortic or cardiac fibroblasts (Figure 6B) reduced the ability of the conditioned media from ANG II–treated cells to induce VSMC growth (Figure 6C and 6D). Taken together, these results suggest that the Nox2-dependent effects of fibroblasts on VSMC growth are mediated to a significant extent by GDF6.

## Discussion

This study identifies a crucial role of fibroblast Nox2 in the development of Ang II–induced vascular remodeling and hypertension. We uncover a novel Nox2-regulated paracrine-signaling mechanism involving the release of GDF6, through which fibroblasts control VSMC growth in the setting of ANG II stimulation. These results strengthen the emerging paradigm that Nox2 has cell-specific functions and its activation contributes to ANG II–induced vascular remodeling and hypertension through distinct molecular mechanisms.

A role for adventitial fibroblasts in regulating vascular functions has been reported in previous work.^23^ Our results are consistent with previous findings that Nox2 contributes to ROS production in mouse aortic fibroblasts but not VSMC.^24^ Another study showed that Nox2 oxidase is expressed in adventitial fibroblasts and may regulate vascular tone by reducing nitric oxide bioavailability.^25^ In addition, fibroblast Nox2 signaling was suggested to influence vascular inflammation and potentially contribute to pathologies such as aortic aneurysm formation.^26^ However, whether fibroblast Nox2 has any specific role in the development of ANG II–induced hypertension and associated vascular remodeling, and what underlying mechanisms may be involved, has been unclear. In the present study, the development and analysis of a new fibroblast-specific Nox2 knockout mouse model allowed us to definitively demonstrate a major role of fibroblast Nox2 in ANG II–induced hypertension.

An increase in peripheral vascular resistance resulting from the remodeling of resistance arteries is recognized to be an important contributing factor to hypertension, both in animal models and humans.^1,2^ The vascular remodeling that occurs during ANG II–induced hypertension is characterized by an increase in medial area and the media/lumen ratio.^8,12,13^ We found that the inhibition of ANG II–induced hypertension in Fibro-Nox2KO mice was accompanied by a substantial reduction in vascular medial thickening and media/lumen ratio, which was observed not only in the aorta but also in smaller resistance vessels in 2 other vascular beds. These findings suggest that the differences in BP between Fibro-Nox2KO and control mice are very likely to be driven by the differences in vascular remodeling. We did not however find any effect of fibroblast Nox2 deficiency on basal BP. These effects contrast to those recently reported in mouse models of endothelial-specific or myeloid cell–specific Nox2 knockout. It was found that myeloid cell Nox2 modulates basal BP through changes in vascular tone but has minimal effects on ANG II–induced hypertension.^16^ However, endothelial cell Nox2 did not affect basal BP but contributed to the early phases (the first 5 days) of ANG II–induced hypertension via ROS-induced changes in vessel tone.^16^ In the current study, it was notable that the effects of fibroblast Nox2 on ANGI II–induced hypertension were most evident towards the end of infusion (after at least 1 week), consistent with a mechanism that involves gradual thickening of the vascular media. Therefore, it is likely that Nox2 in different cell types contributes to ANG II–induced hypertension in mice through different mechanisms that are effective at different stages of the development of hypertension in this model. Interestingly, a previous study that conditionally targeted AT1 (angiotensin type 1) receptors in different cell types in mice in vivo reported that ANG II–induced aortic medial thickening involves AT1 receptors on adventitial fibroblasts rather than on VSMC or endothelial cells.^27^ The current findings in the Fibro-Nox2KO mice are, therefore, consistent with these previous results and support a fibroblast-mediated mechanism for vascular remodeling induced by ANG II. The precise relationship between the vascular remodeling and hypertension or the longer-term impact of the changes in vascular remodeling on ANG II–induced hypertension was not investigated in our study and therefore remain speculative.

To investigate the mechanism by which fibroblast Nox2 affects remodeling of the vascular media, we hypothesized that it involves a paracrine effect of fibroblasts on VSMC growth. Consistent with this notion, there was evidence of significantly increased medial VSMC growth in the aorta after chronic ANG II infusion in control mice, which was not observed in Fibro-Nox2KO animals. Furthermore, the conditioned medium of ANG II–stimulated cultured fibroblasts induced a significant increase in VSMC growth which was Nox2- and ROS-dependent. This action did not involve the direct effects of hydrogen peroxide diffusing from fibroblasts to VSMC. Using a proteomic analysis of the secretome of ANG II–stimulated cultured fibroblasts along with bioinformatic analysis, we homed down on a small number of putative fibroblast-specific candidate factors that might account for the paracrine effect of fibroblast-secreted factors on VSMC growth. Among these, the TGF-β superfamily member GDF6 was a likely candidate given that it was regulated in a Nox2 and ROS-dependent manner in the aorta, as well as fibroblasts in vitro. Indeed, we found evidence of Nox2-dependent release of GDF6 into the secretome of ANG II–stimulated fibroblasts as well as increased GDF6 protein levels and immunoreactivity in the aorta of control mice stimulated with ANG II but not Fibro-Nox2KO animals. Finally, recombinant GDF6 significantly increased VSMC growth in vitro, and the silencing of GDF6 in fibroblasts prevented the effects of fibroblast-conditioned media on VSMC.

GDF6 is a member of the TGF-β superfamily of proteins and is known to be involved in regulating ocular and skeletal development in multiple species including humans.^28–31^ Eye disorders that are linked to GDF6 mutations include microphthalmia and age-related macular degeneration.^29,31^ Abnormalities of skeletal development linked to GDF6 mutations include vertebral fusion and abnormal joint development, for example, in the Klippel-Feil syndrome.^30^ Very little is known about the function of GDF6 in the cardiovascular system apart from a very recent report suggesting a role in vascular stabilization in zebrafish.^32^ However, as a member of the TGF-β superfamily that is reported to signal through the phosphorylation of SMAD (similar to mothers against decapentaplegic) transcription factors,^33^ a growth role is predictable. Other TGF-β superfamily members such as TGF-β1-3 have been implicated in vascular remodeling but we did not detect significant levels of TGF-β in the conditioned medium of ANG II–treated fibroblasts, although mRNA levels were reduced in fibroblasts after knockdown of Nox2. Nevertheless, we cannot discount the possibility that the fibroblast-mediated enhancement of vascular medial thickening observed in the current study may involve the action of GDF6 in combination with other secreted factors. It is also possible that Nox2-dependent inactivation of endothelial nitric oxide could synergize with such effects given that nitric oxide has inhibitory effects on VSMC growth.^34^ The mechanism by which Nox2 increases the release of GDF6 from fibroblasts requires further study but appears to be transcriptional and redox-dependent because we found an inhibition of the ANG II–induced increase in GDF6 mRNA levels when Nox2 was knocked down or the cells were incubated with superoxide dismutase and catalase.

In summary, this study identifies a novel paracrine-signaling mechanism involving fibroblast Nox2-dependent secretion of GDF6, which promotes vascular remodeling and hypertension in response to chronic ANG II elevation (Graphic abstract). The timing of effect of fibroblast Nox2 on ANG II–induced hypertension contrasts to the effects of endothelial Nox2 studied in the same model, which occur earlier and are related to altered vascular tone.^16^ Our results add to the emerging concept of Nox2 as a master regulator of ANG II–induced hypertension and also suggest that GDF6 could be a potential target to inhibit vascular remodeling during increased activation of the renin-angiotensin system.

## Sources of Funding

Supported by the British Heart Foundation (RG/13/11/30384 [A.M. Shah], CH/1999001/11735 [A.M. Shah], CH/16/3/32406 [M. Mayr], RG/16/14/32397 [M. Mayr] and PG/17/48/32956 [M. Mayr]).

## Disclosures

None.

## Supplementary Material


