# Supplementary material for: Fibroblast Nox2 (NADPH Oxidase-2) Regulates ANG II (Angiotensin II)–Induced Vascular Remodeling and Hypertension via Paracrine Signaling to Vascular Smooth Muscle Cells
Source: Arterioscler Thromb Vasc Biol. 2020 Oct 15;41(2):698–710. doi: 10.1161/ATVBAHA.120.315322 (PMC7837692; doi:10.1161/ATVBAHA.120.315322)
Supplement: Supplementary file 1 [file atv-41-698-s001.pdf]

SUPPLEMENTAL MATERIALS

Fibroblast Nox2 regulates angiotensin II-induced vascular remodeling and hypertension via paracrine signaling to vascular smooth muscle cells

Craig B. Harrison PhD[1], Silvia Cellone Trevelin PhD[1], Daniel A. Richards PhD[1], Celio X.C. Santos PhD[1], Greta Sawyer PhD[1], Andrea Markovinovic PhD[2], Xiaohong Zhang MD[1], Min Zhang MD, PhD[1], Alison C. Brewer PhD[1], Xiaoke Yin PhD[1], Manuel Mayr MD PhD[1], Ajay M. Shah MD[1], FMedSci.

[1]King's College London British Heart Foundation Centre of Excellence, School of Cardiovascular Medicine and Sciences, London, United Kingdom.[2]King's College London, Department of Basic and Clinical Neuroscience, Maurice Wohl Clinical Neuroscience Institute, London, United Kingdom.

Correspondence to: Professor Ajay M Shah, King's College London BHF Centre of Excellence, James Black Centre, 125 Coldharbour Lane, London SE5 9NU, UK
Tel: 44-2078485189; Fax: 44-2078485193; Email: ajay.shah@kcl.ac.uk

Contents

Supplemental Figure I. mRNA expression levels of Nox2 regulatory subunits and Nox isoforms in Fibro-Nox2KO and control Nox2Flox aorta.

Supplemental Figure II. Fibroblast-specific targeting of Nox2.

Supplemental Figure III. Effect of fibroblast-specific Nox2 deletion on ROS levels.

Supplemental Figure IV. Effect of fibroblast-specific Nox2 deletion on renal function.

Supplemental Figure V. Nox2 protein levels in subfornical organ of the Fibro-Nox2KO mouse brain.

Supplemental Figure VI. Effect of conditioned medium of aortic fibroblasts stimulated with ANG II on DNA synthesis of VSMC.

Supplemental Figure VII. Effect of catalase treatment of fibroblast-conditioned medium.

Supplemental Figure VIII. Proteomic analyses of the conditioned medium of primary cardiac fibroblasts.

Supplemental Figure IX. Proteomic analyses of the conditioned medium of cardiac fibroblasts.

Supplemental Figure X. Effect of ANG II on mRNA expression of putative fibroblast growth factors.

Supplemental Figure XI. HTRA1 protein levels in fibroblast-conditioned medium.

Supplemental Figure XII. ELISA for TGFβ in fibroblast-conditioned medium.

Source immunoblots

Major Resources Table

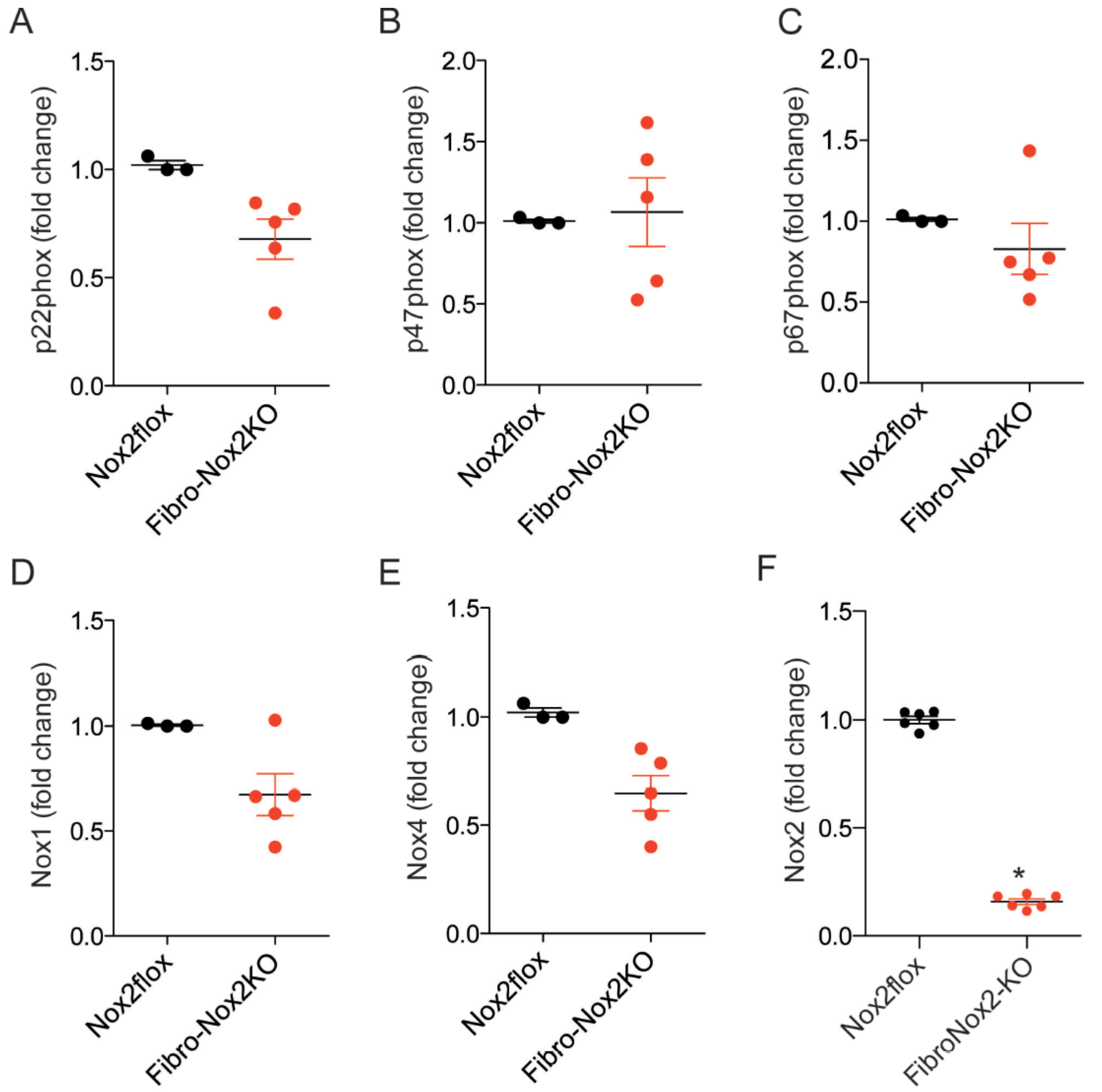

Supplemental Fig. I. mRNA expression levels of Nox2 regulatory subunits and Nox isoforms in Fibro-Nox2KO and control Nox2Flox aorta. n=3-6/group. *P<0.05, unpaired Student's t-test.

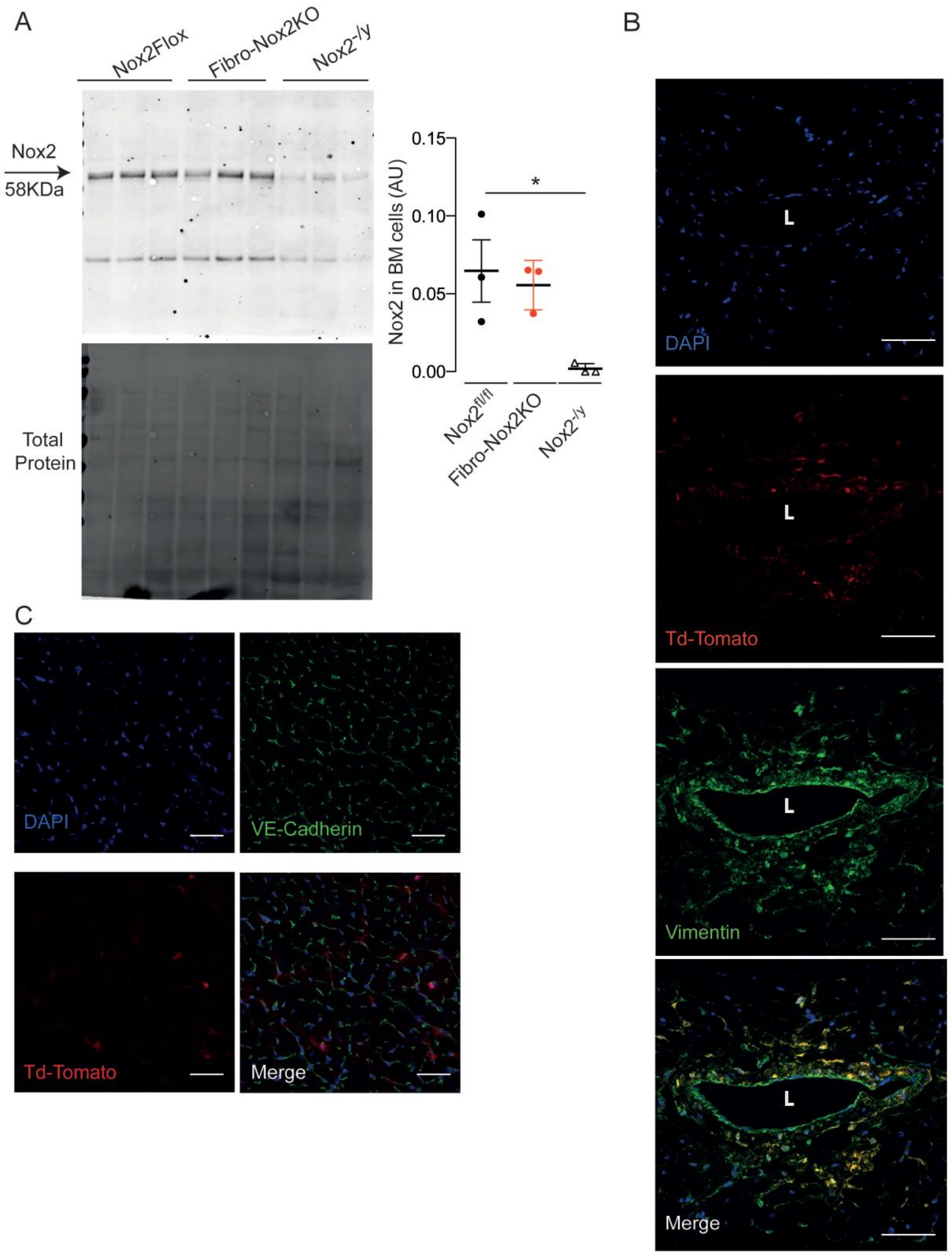

Supplemental Fig. II. Fibroblast-specific targeting of Nox2. (A) Nox2 protein levels are unaltered in bone marrow cells of FibroNox2KO mice by immunoblotting. The levels of Nox2 were normalized by total protein. AU, arbitrary units. n=3 per group. *P<0.05; Kruskal-Wallis followed by Dunn's post-test. (B) Coronary vessel in myocardial section of *Col1α2*CreER:ROSA26R-tdTomato$^{fl/fl}$ mice. Green, vimentin; Red, Td-Tomato; Blue, DAPI; L, lumen. Td-Tomato overlaps with vimentin in the adventitia but not at the endothelium. (C) Myocardial sections stained for EC with anti-VE-Cadherin Ab (green). Red, Td-Tomato; Blue, DAPI. There is no overlap between Td-Tomato and VE-cadherin. Scale bars, 50 µm.

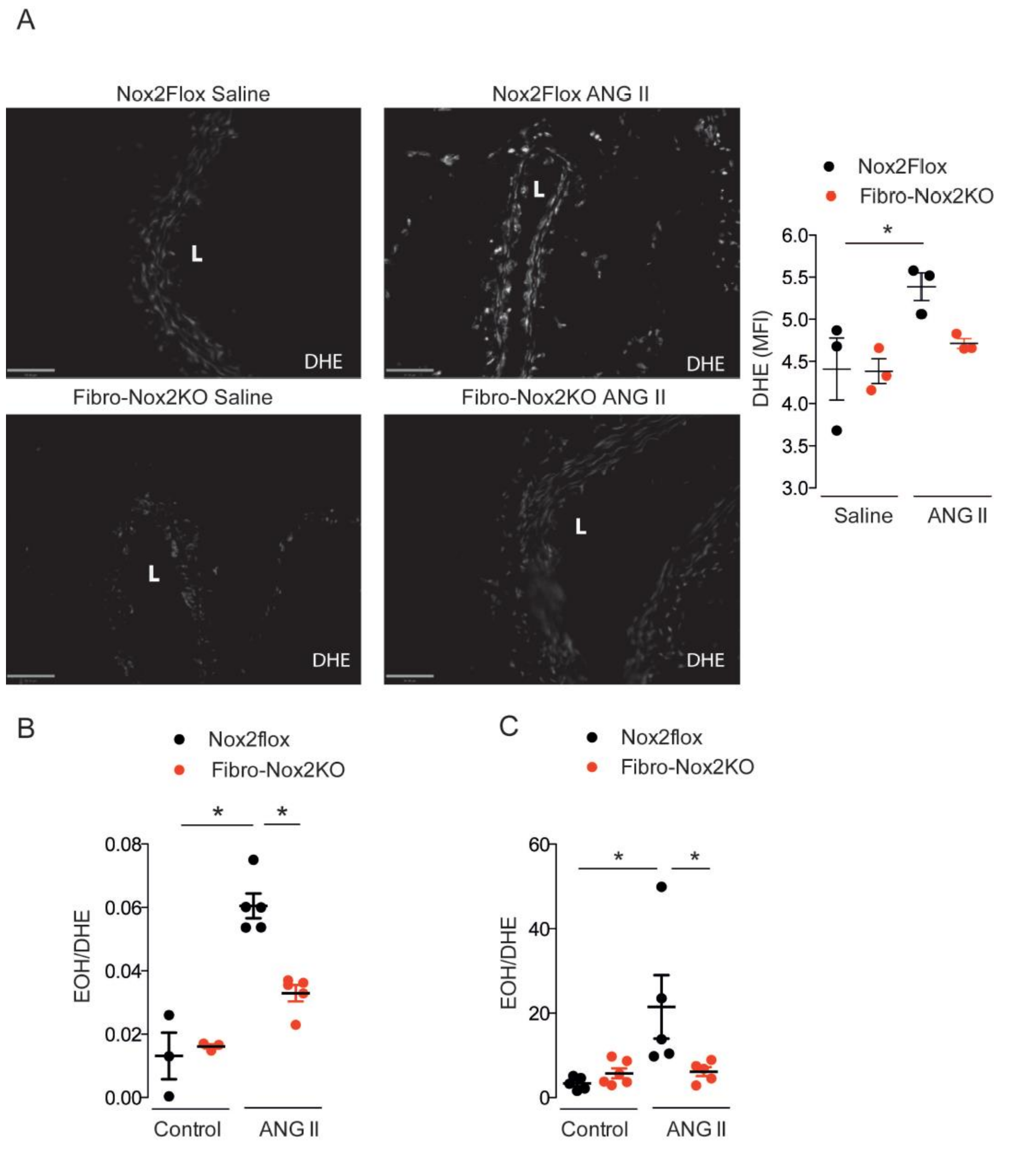

Supplemental Fig. III. Effect of fibroblast-specific Nox2 deletion on ROS levels. (A) Dihydroethidium (DHE) fluorescence as an index of ROS levels in aorta of Fibro-Nox2KO and Nox2Flox mice after 14 days of ANG II or saline infusion. Representative images are shown to the left and mean data to the right (n=3/group). L, lumen; MFI, mean fluorescence intensity. Scale bars, 50µm. (B) Levels of DHE oxidation product 2-hydroxyethidine (EOH) expressed as a ratio of the DHE consumed, and normalized by aortic segment size. EOH and DHE were quantified by HPLC using area under curve. Aortic segments were obtained from Fibro-Nox2KO or Nox2flox control mice infused with ANG II or saline. (C) EOH levels quantified as in B, using fibroblasts stimulated with ANG II. n=3-6 per group for B and C. *P<0.05; Kruskal-Wallis followed by Dunn's post-test (A) and One-way ANOVA followed by Tukey post-test (B, C).

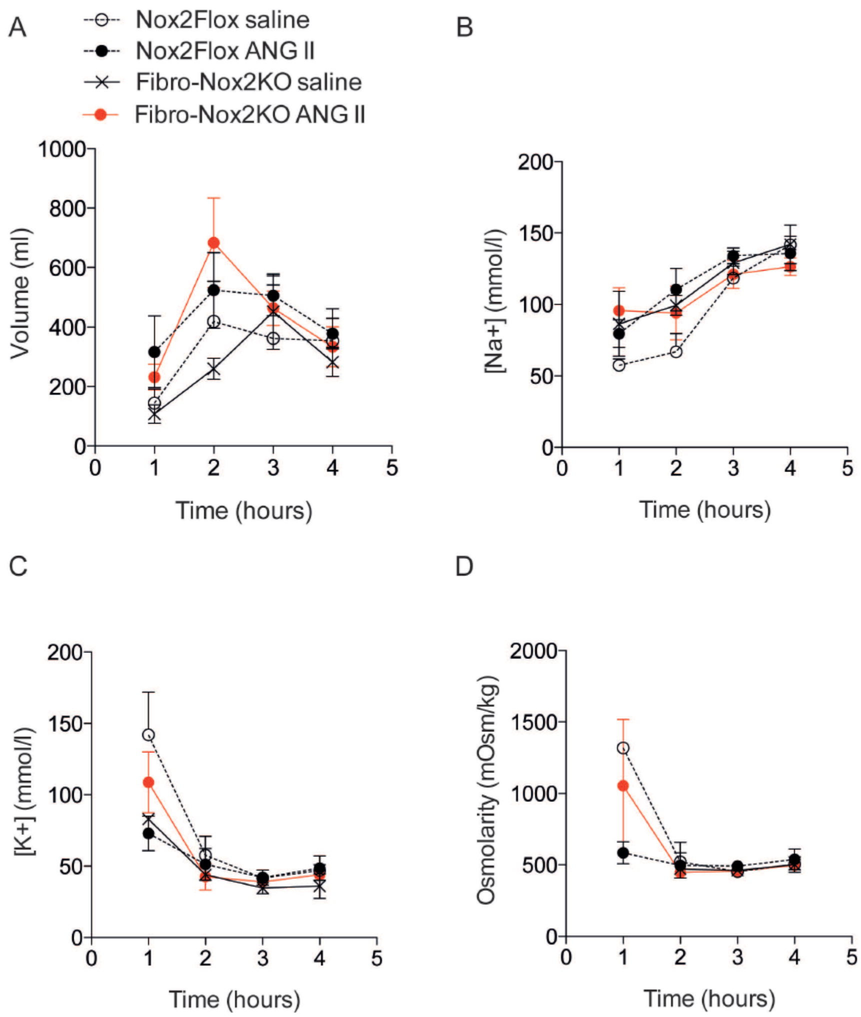

Supplemental Fig. IV. Effect of fibroblast-specific Nox2 deletion on renal function. Following an intraperitoneal injection of PBS, urine was analysed for (A) volume, (B) sodium concentration, (C) potassium concentration, and (D) osmolarity. n=3/group.

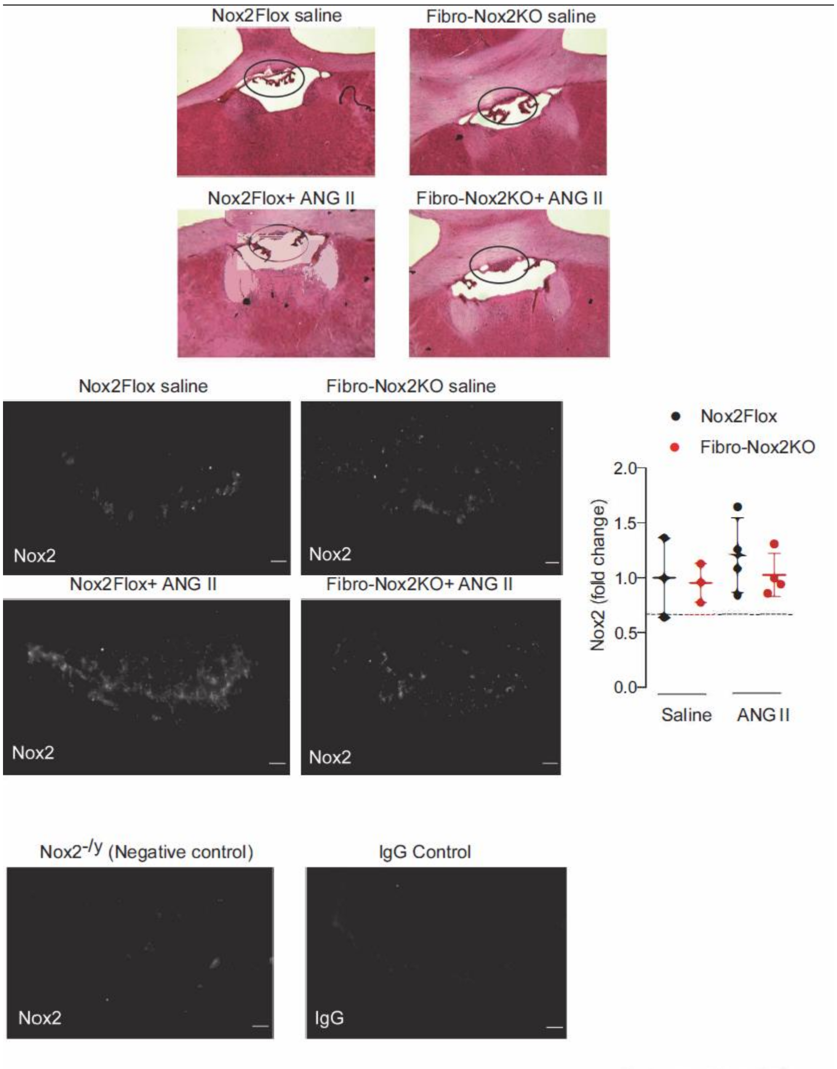

Supplemental Fig. V. Nox2 protein levels in subfornical organ of the Fibro-Nox2KO mouse brain. Fibro-Nox2KO and Nox2Flox mice were treated for 14 days with ANG II infusion or saline and the brain was sectioned. The top 4 panels show brain sections stained with H&E; the subfornical organ (SFO) is indicated by the area circled in black. Immunofluorescence for Nox2 protein in the SFO was assessed using a Nox2 Ab. Brain sections from Nox2$^{-/y}$ (global KO) and Nox2Flox mice treated with ANG II were incubated with IgG instead of Nox2Ab as negative controls. Scale bars: 20 μm. The fold change in mean fluorescence intensity is shown on the right. Dashed line represents the mean signal in Nox2$^{-/y}$ mice relative to Nox2flox mice.

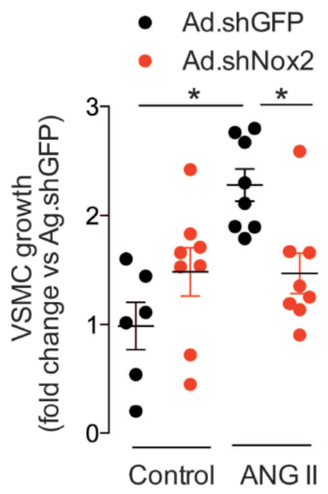

Supplemental Fig. VI. Effect of conditioned medium of aortic fibroblasts stimulated with ANG II on DNA synthesis of VSMC. DNA synthesis was assessed by Ki67 staining. n=6-8/group. *P<0.05; One-way ANOVA followed by Tukey post-test.

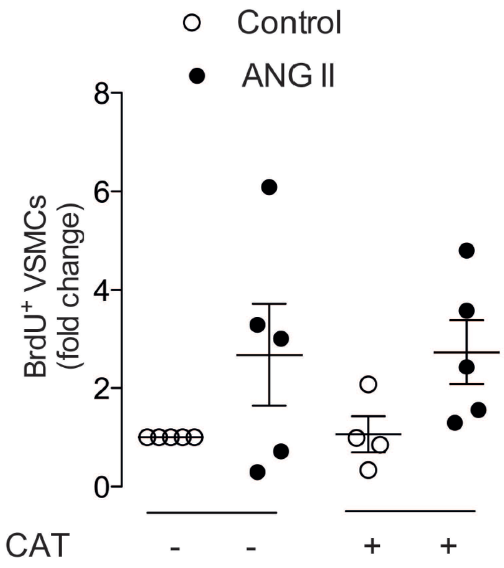

Supplemental Fig. VII. Effect of catalase treatment of fibroblast-conditioned medium. Catalase treatment does not inhibit the effect of cardiac fibroblast-conditioned medium on VSMC growth (assessed by BrdU incorporation). n=4-5/group.

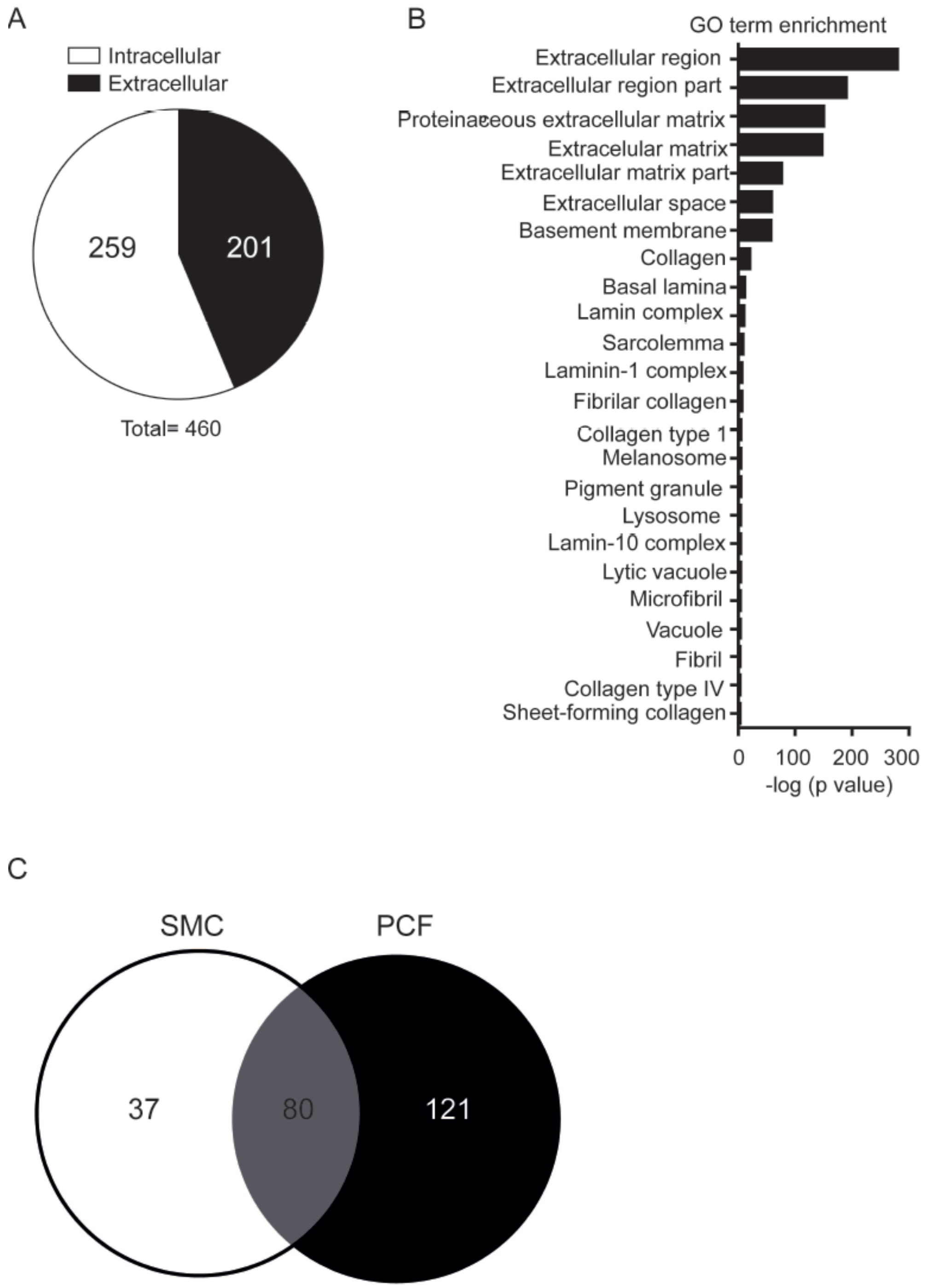

Supplemental Fig. VIII. Proteomic analyses of the conditioned medium of primary cardiac fibroblasts. (A) Proportions of intracellular versus extracellular proteins identified in all samples. (B) Gene ontology (GO) term enrichment to identify putative extracellular or secreted proteins. (C) Extracellular/secreted proteins identified in the fibroblast conditioned medium (PCF) that are distinct from those found in the mouse VSMC secretome (SMC).

Accession Number	Accession Number	Accession Number	Accession Number
LAMA4_MOUSE	LYZ2_MOUSE	APOE_MOUSE	LKHA4_MOUSE
LAMB2_MOUSE	TETN_MOUSE	CBPQ_MOUSE	TCPZ_MOUSE
LAMA2_MOUSE	AK1A1_MOUSE	MFAP4_MOUSE	KCY_MOUSE
CO5A1_MOUSE	ATS2_MOUSE	SEM3D_MOUSE	DPEP1_MOUSE
LUM_MOUSE	CS1B_MOUSE	K1199_MOUSE	DOPD_MOUSE
POSTN_MOUSE	COMP_MOUSE	CFAB_MOUSE	TOIP1_MOUSE
CO8A1_MOUSE	ATL2_MOUSE	RLA2_MOUSE	SFRP1_MOUSE
CTGF_MOUSE	GDN_MOUSE	CXCL5_MOUSE	MGP_MOUSE
CATD_MOUSE	LOXL2_MOUSE	DKK3_MOUSE	MA2B2_MOUSE
TRFE_MOUSE	SFRP3_MOUSE	OAF_MOUSE	INHBA_MOUSE
ELN_MOUSE	PRDX2_MOUSE	ATS5_MOUSE	BGH3_MOUSE
SPA3N_MOUSE	FRIL1_MOUSE	GGH_MOUSE	SEM3C_MOUSE
TPM1_MOUSE	MFAP5_MOUSE	IQGA1_MOUSE	LBP_MOUSE
MMP12_MOUSE	SRPX2_MOUSE	QPCT_MOUSE	NGAL_MOUSE
LOXL1_MOUSE	PROS_MOUSE	CCL7_MOUSE	LGUL_MOUSE
FBLN1_MOUSE	EF1G_MOUSE	LYZ1_MOUSE	PEPD_MOUSE
HPT_MOUSE	MFGM_MOUSE	HEG1_MOUSE	TFPI1_MOUSE
TCO2_MOUSE	ANXA5_MOUSE	TPIS_MOUSE	GILT_MOUSE
COEA1_MOUSE	ISLR_MOUSE	PLTP_MOUSE	ADML_MOUSE
CERU_MOUSE	APOD_MOUSE	NEUS_MOUSE	RISC_MOUSE
TKT_MOUSE	VEGFD_MOUSE	CREG1_MOUSE	PLAC9_MOUSE
CO4B_MOUSE	COFA1_MOUSE	EPDR1_MOUSE	GROA_MOUSE
WISP2_MOUSE	PRELP_MOUSE	NUCB2_MOUSE	ALBU_MOUSE
FBLN3_MOUSE	SRCRL_MOUSE	XDH_MOUSE	ANGL2_MOUSE
DERM_MOUSE	CCL2_MOUSE	SPON2_MOUSE	SLIT2_MOUSE
SFRP2_MOUSE	ITGBL_MOUSE	BTD_MOUSE	ASM3A_MOUSE
TAGL2_MOUSE	ATS1_MOUSE	DIAC_MOUSE	HTRA1_MOUSE
IBP2_MOUSE	FRIH_MOUSE	COGA1_MOUSE	CPXM1_MOUSE
OLFL3_MOUSE	SPRL1_MOUSE	GDF6_MOUSE	OSTP_MOUSE
SH3L1_MOUSE	LTBP2_MOUSE	PRS23_MOUSE	IBP5_MOUSE

Supplemental Fig. IX. Proteomic analyses of the conditioned medium of cardiac fibroblasts. Gene ontology (GO) enrichment analysis of 121 fibroblast-specific secreted proteins to identify putative growth factors (highlighted).

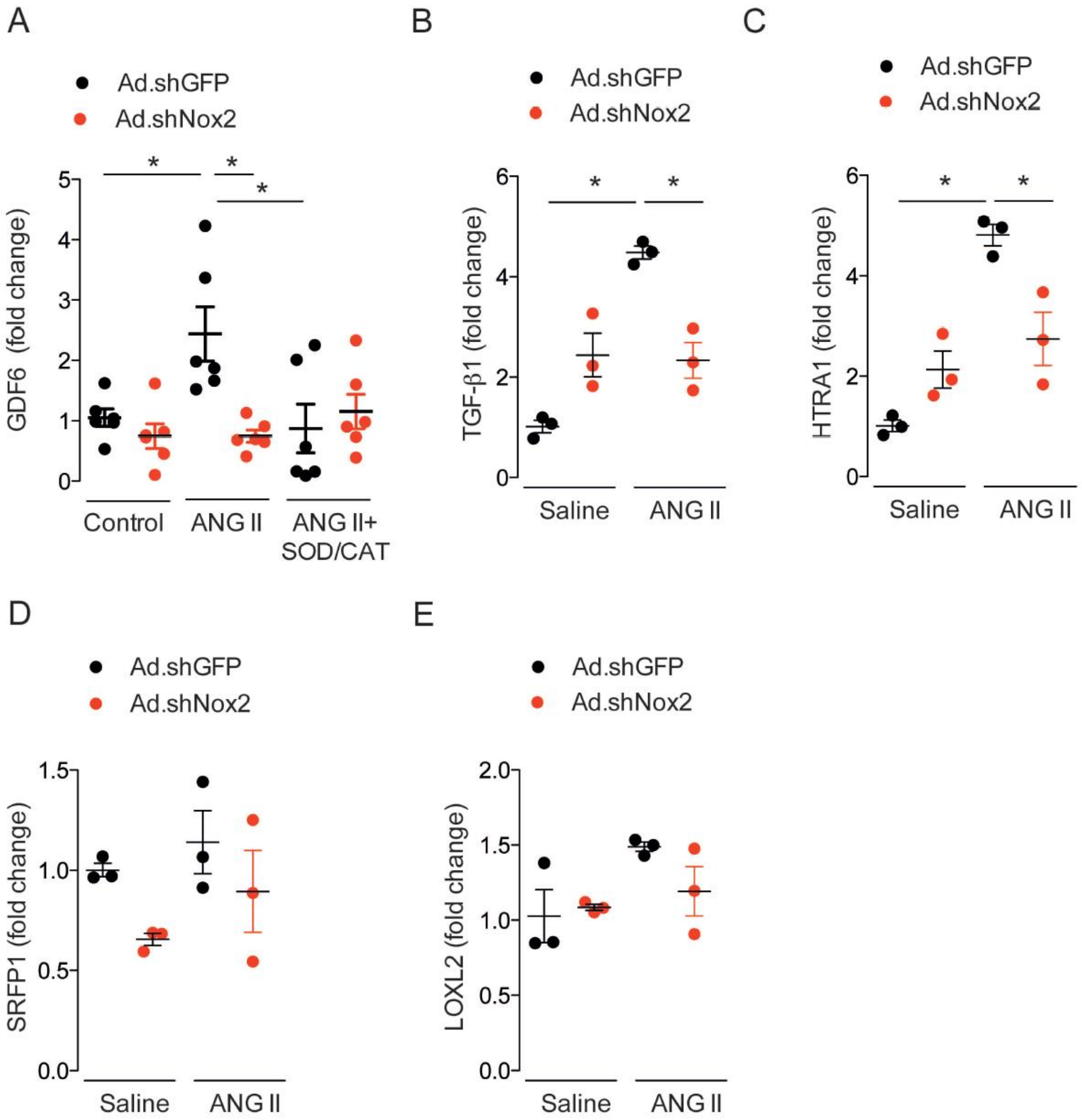

Supplemental Fig. X. Effect of ANG II on mRNA expression of putative fibroblast growth factors. GDF6, TGF-β1, HTRA1, SRFP1 and LOXL2 mRNA were quantified in Nox2-deficient (Ad-shNox2) and control cardiac fibroblasts (Ad-shGFP). Some cells were pre-incubated with PEG-SOD plus PEG-CAT before stimulation with ANG II. *$P<0.05$; Kruskal-Wallis followed by Dunn's post-test.

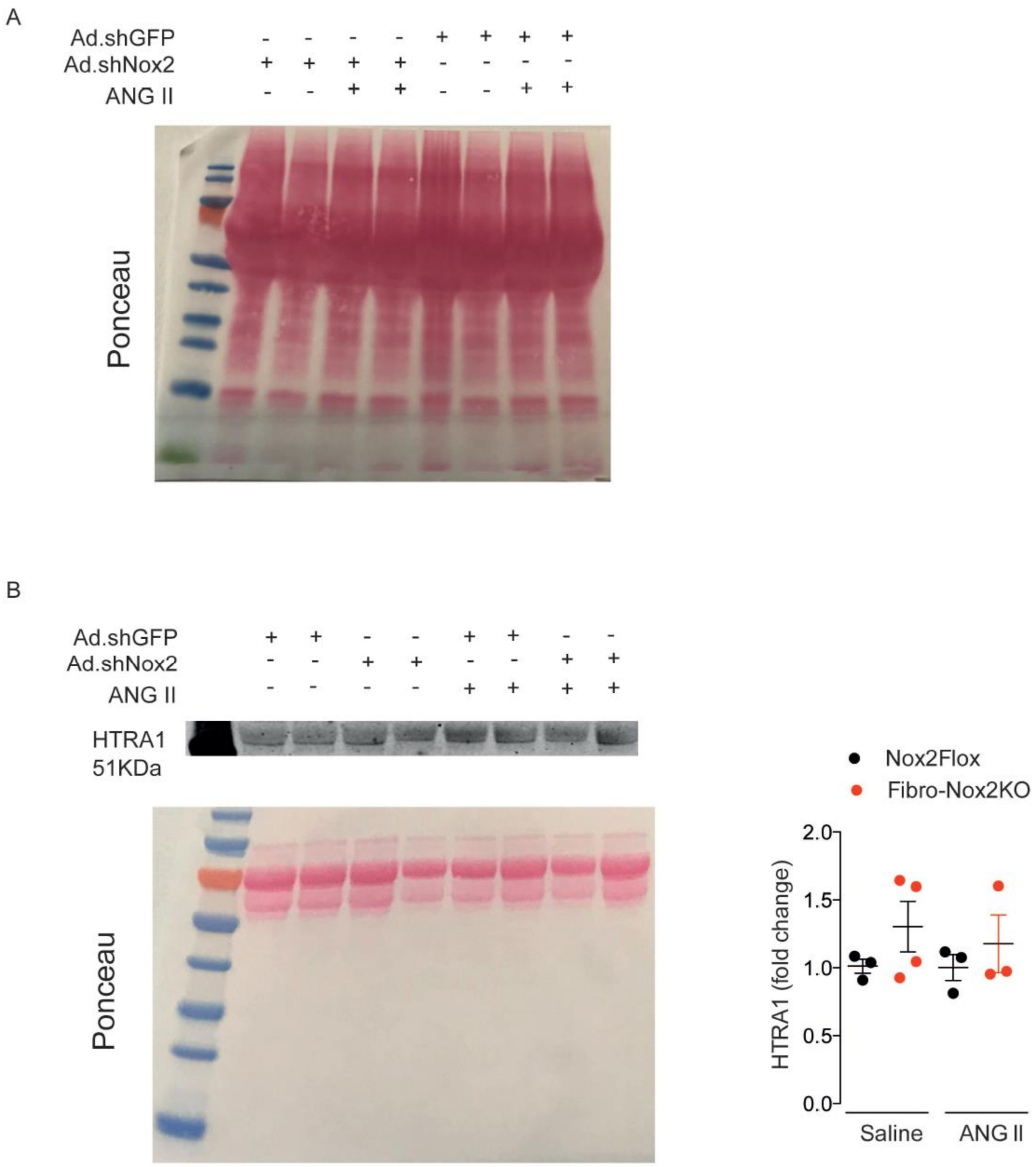

Supplementary Fig. XI. HTRA1 protein levels in fibroblast-conditioned medium. A. Ponceau staining of the membrane used in GDF6 immunoblot in Figure 5A. B. HTRA1 levels in conditioned medium of primary cardiac fibroblasts infected with Ad.shNox2 or Ad.shGFP (control) and treated with ANG II. Representative immunoblot and Ponceau stained membrane are shown to the left and mean data to the right; n=3/group.

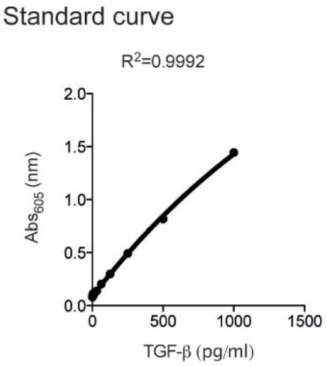

	Abs 605nm (mean± SD)
Ad.GFP	0.05± 0.003
Ad.GFP+ ANG II	0.06± 0.001
Ad.Nox2	0.06± 0.006
Ad.Nox2+ ANG II	0.05± 0.002

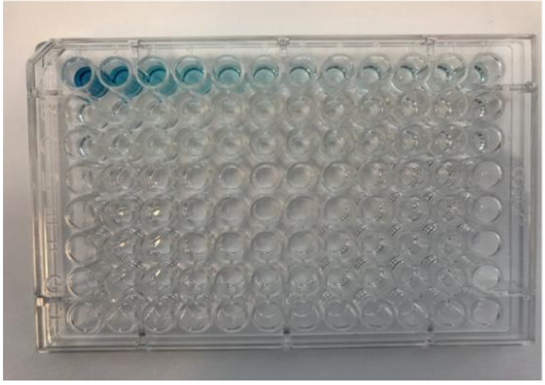

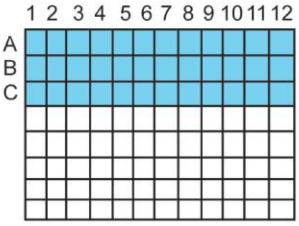

A1-12 Standard curve
B1-4 Ad.GFP
B5-12 Ad.GFP+ ANG II
C1-4 Ad.Nox2
C5-12 Ad.Nox2+ ANG II

Supplementary Fig. XII. ELISA for TGFβ in fibroblast-conditioned medium. Top left panel shows the standard curve for the assay. Top right are the results of the assay. The image and schematic at the bottom show the layout of samples on the ELISA plate. Cardiac fibroblasts were infected with Ad.shNox2 or Ad.shGFP (control) and treated with ANG II. n=3/group.

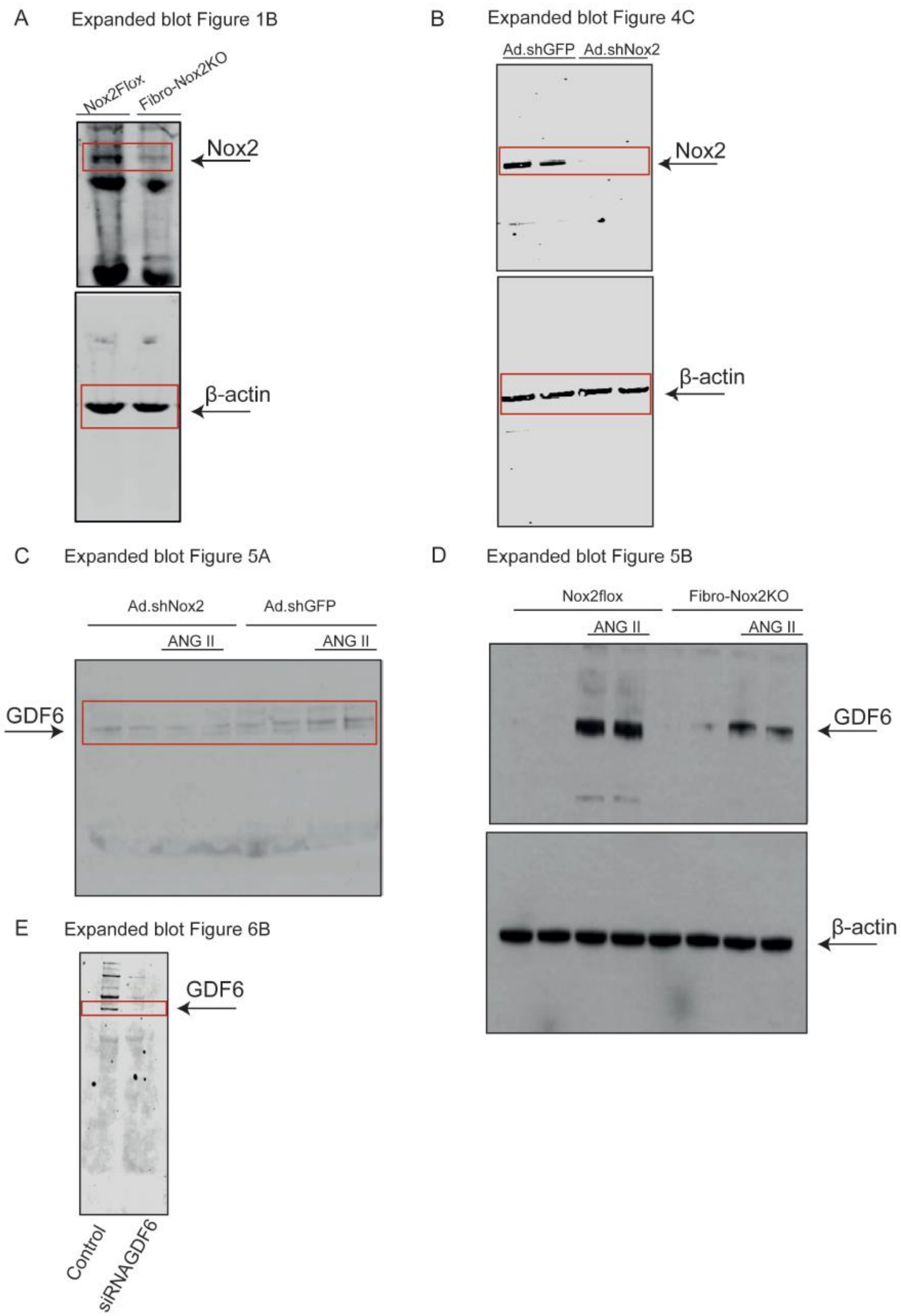

Complete source blots. Source blots relate to the figures indicated in the panel headings.

Major Resources Table

In order to allow validation and replication of experiments, all essential research materials listed in the Methods should be included in the Major Resources Table below. Authors are encouraged to use public repositories for protocols, data, code, and other materials and provide persistent identifiers and/or links to repositories when available. Authors may add or delete rows as needed.

Animals (in vivo studies)

Species	Vendor or Source	Background Strain	Sex	Persistent ID / URL
Nox2flox	Local KCL BSU	C57BL/6J	M	
FibroNox2KO	Local KCL BSU	C57BL/6J	M	
Cre-Td+Nox2fl/fl	Local KCL BSU	C57BL/6J	M	
Nox2y/-	Local KCL BSU	C57BL/6J	M	

Genetically Modified Animals

	Species	Vendor or Source	Background Strain	Other Information	Persistent ID / URL
**Parent - Male**	*Col1α2*CreER-Tg	Local KCL BSU	C57BL/6J		
**Parent - Female**	Nox2flox	Local KCL BSU	C57BL/6J		

Antibodies

Target antigen	Vendor or Source	Catalog #	Working concentration	Lot # (preferred but not required)	Persistent ID / URL
Nox2	BD Biosciences	611415	2.5μg/ml for IF and 0.25 μg/ml for WB		
GDF6	Abcam	ab73288	10μg/ml for IF and 1μg/ml for WB		
HTRA1	Abcam	ab36611	2μg/ml		
Alpha-smooth muscle actin	Abcam	A2447	4μg/ml		
Ki67	Novus Biological	NB-600 1252	0.5μL (1:200)	1030	https://www.novusbio.com/PDFs/NB600-1252.pdf
Vimentin	Abcam	ab8069	4μg/ml		
Beta-actin	Sigma	a2228	0.67μg/ml		
VE-cadherin	Biolegend	138002	10μg/ml		
BrDU	Abcam	ab152095	10μg/ml		

DNA/cDNA Clones

Clone Name	Sequence	Source / Repository	Persistent ID / URL

Cultured Cells

Name	Vendor or Source	Sex (F, M, or unknown)	Persistent ID / URL
C57Bl/6 mouse primary fibroblast	Cell Biologics (Cat. C57-6075)	Unknown	https://www.cellbiologics.net/document/data-sheet/Mouse/WT-MS/C57-cells-DONE/C57%20FB/C57-6075.pdf

Data & Code Availability

Description	Source / Repository	Persistent ID / URL

Other

Description	Source / Repository	Persistent ID / URL
