# Supplementary figures and images for: Fibroblast Nox2 (NADPH Oxidase-2) Regulates ANG II (Angiotensin II)–Induced Vascular Remodeling and Hypertension via Paracrine Signaling to Vascular Smooth Muscle Cells
Source: Arterioscler Thromb Vasc Biol. 2020 Oct 15;41(2):698–710. doi: 10.1161/ATVBAHA.120.315322 (PMC7837692; doi:10.1161/ATVBAHA.120.315322)

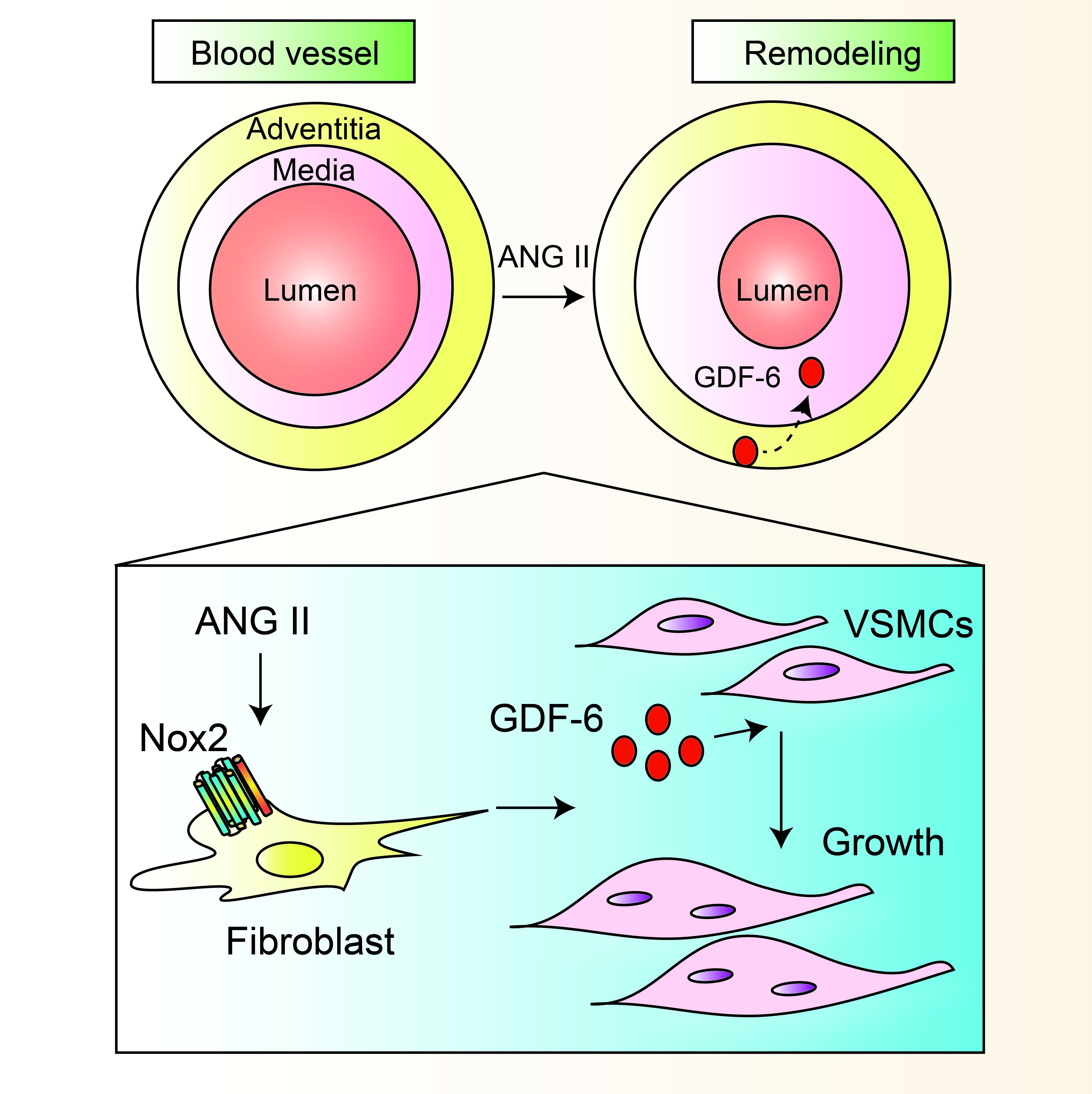

Supplement: Supplementary file 2 [file atv-41-698-s002.jpg]
